# RetiGene, a comprehensive gene atlas for inherited retinal diseases

**DOI:** 10.1016/j.ajhg.2025.08.017

**Published:** 2025-09-16

**Authors:** Carlo Rivolta, Elifnaz Celik, Dhryata Kamdar, Francesca Cancellieri, Karolina Kaminska, Mukhtar Ullah, Pilar Barberán-Martínez, Manon Bouckaert, Marta Cortón, Emma Delanote, Lidia Fernández-Caballero, Gema García García, Lara K. Holtes, Marianthi Karali, Irma Lopez, Virginie G. Peter, Nina Schneider, Lieselot Vincke, Carmen Ayuso, Sandro Banfi, Beatrice Bocquet, Frauke Coppieters, Frans P.M. Cremers, Chris F. Inglehearn, Takeshi Iwata, Vasiliki Kalatzis, Robert K. Koenekoop, José M. Millán, Dror Sharon, Carmel Toomes, Mathieu Quinodoz

**Affiliations:** 1 Ophthalmic Genetics Group, Institute of Molecular and Clinical Ophthalmology Basel (IOB), Basel 4031, Switzerland; 2 Department of Ophthalmology, University of Basel, Basel 4031, Switzerland; 3 Department of Genetics, Genomics and Cancer Sciences, University of Leicester, Leicester LE1 7RH, UK; 4 Molecular, Cellular, and Genomic Biomedicine Group, IIS-La Fe, Valencia 46026, Spain; 5 Joint Unit CIPF-IIS La Fe Molecular, Cellular and Genomic Biomedicine, IIS-La Fe, Valencia 46026, Spain; 6 Center for Medical Genetics Ghent, Ghent University, Ghent 9000, Belgium; 7 Department of Biomolecular Medicine, Ghent University, Ghent 9000, Belgium; 8 Department of Genetics & Genomics, Instituto de Investigación Sanitaria-Fundación Jiménez Díaz University Hospital, Universidad Autónoma de Madrid (IIS-FJD, UAM), Madrid 28040, Spain; 9 Center for Biomedical Network Research on Rare Diseases (CIBERER), Instituto de Salud Carlos III, Madrid 28029, Spain; 10 Department of Human Genetics, Radboud University Medical Center, Nijmegen, 6525 GA, the Netherlands; 11 Department of Precision Medicine, Medical Genetics, Università degli Studi della Campania "Luigi Vanvitelli", Naples 80138, Italy; 12 Multidisciplinary Department of Medical, Surgical and Dental Sciences, Eye Clinic, Università degli Studi della Campania "Luigi Vanvitelli", Naples 80138, Italy; 13 Department of Paediatric Surgery, Human Genetics, and Ophthalmology, McGill Ocular Genetics Laboratory and Centre, McGill University, Montreal, QC H4A 3S5, Canada; 14 Department of Ophthalmology, Bern University Hospital, Bern 3010, Switzerland; 15 Department of Ophthalmology, Hadassah Medical Center, The Hebrew University of Jerusalem, Jerusalem 91120, Israel; 16 Telethon Institute of Genetics and Medicine, Pozzuoli 80078, Italy; 17 Institute for Neurosciences of Montpellier, Université de Montpellier, Montpellier 34091, France; 18 Department of Pharmaceutics, Ghent University, Ghent 9000, Belgium; 19 Leeds Institute of Medical Research, Division of Molecular Medicine, University of Leeds, Leeds LS2 9JT, UK; 20 Division of Molecular and Cellular Biology, National Institute of Sensory Organs, NHO Tokyo Medical Center, Tokyo 152-8902, Japan

**Keywords:** IRD, inherited retinal diseases, database

## Abstract

Inherited retinal diseases (IRDs) are rare disorders, typically presenting as Mendelian traits, that result in stationary or progressive visual impairment. They are characterized by extensive genetic heterogeneity, possibly the highest among all human genetic diseases, as well as diverse inheritance patterns. Despite advances in gene discovery, limited understanding of gene function and challenges in accurately interpreting variants continue to hinder both molecular diagnosis and genetic research in IRDs. One key problem is the absence of a comprehensive and widely accepted catalog of disease-associated genes, which would ensure consistent genetic testing and reliable molecular diagnoses. With the rapid pace of IRD gene discovery, gene catalogs require frequent validation and updates to remain clinically and scientifically useful. To address these gaps, we developed RetiGene, an expert-curated gene atlas that integrates variant data, bulk and single-cell RNA sequencing, and functional annotations. Through the integration of diverse data sources, RetiGene supports candidate gene prioritization, functional studies, and therapeutic development in IRDs.

## Teaser

RetiGene is an expert-curated atlas of genes involved in inherited retinal diseases, integrating variant data and gene expression. This open-access, continually updated resource aims to support variant interpretation, gene panel design, and future therapeutic research.

## Introduction

The retina is a photosensitive tissue lining the posterior part of the eye. Its primary function is to convert light into electrical signals, which are then transmitted to the brain to form visual images. The retina contains two types of photoreceptors: rods and cones. Rods are responsible for vision in low-light conditions, while cones provide sharp central vision, enable color perception, and mediate sight in bright-light environments.^1^ The retinal pigment epithelium (RPE), a layer of pigmented cells located between the photoreceptors and the choroid, plays a crucial role in supporting vision. It absorbs excess light, forms part of the blood-retina barrier, transports nutrients and waste, regulates the visual cycle, and removes photoreceptor debris, thereby ensuring their proper function.^2^^,^^3^ Other retinal cell types include retinal ganglion cells, horizontal cells, amacrine cells, and Müller cells, among others, whose function is to encode visual signals detected by photoreceptors and to ensure the correct homeostasis of the retina by providing structural, metabolic, and immunological support.^4^^,^^5^^,^^6^^,^^7^

Inherited retinal diseases (IRDs) are a diverse group of monogenic conditions that typically lead to the progressive degeneration or dysfunction of photoreceptors, RPE cells, or other retinal neurons, culminating in vision loss and, in many cases, blindness. Clinically, IRDs are categorized based on the cell types that are first or predominantly affected (e.g., rod-cone degeneration, cone dystrophy, cone-rod degeneration, etc.), the portion of the retina that is primarily involved (center vs. periphery, such as in Stargardt disease and retinitis pigmentosa [RP], respectively), and/or the presence of disease progression (stationary vs. progressive).^8^^,^^9^ They can also be further classified as non-syndromic (affecting only the eye) or syndromic (affecting the eye along with other organs, such as the auditory or renal systems).^10^

RP is the most prevalent form of IRD, characterized by the degeneration of rods, primarily, and cones, at a later stage,^11^^,^^12^ whereas cone and cone-rod dystrophies (CDs/CRDs) are characterized by the exclusive or primary loss of cones, respectively.^13^^,^^14^ The generally stationary forms of cone disorders, grouped as color vision disorders (CVDs), include achromatopsia, blue-cone monochromacy, and common color blindness.^15^ Similarly, congenital stationary night blindness (CSNB) is the non-progressive form of rod dysfunction.^16^ Macular diseases (MDs), in which degeneration is largely restricted to the macula, include the second most prevalent form of IRDs, Stargardt disease, as well as Best disease, Sorsby macular dystrophy, etc.^12^^,^^17^^,^^18^ Lastly, some other non-syndromic IRDs indirectly affect photoreceptors or involve other retinal cell types, such as optic atrophies (OAs), exudative vitreoretinopathies (EVRs), etc.^19^^,^^20^ The most severe form of non-syndromic IRDs is Leber congenital amaurosis (LCA), characterized by retinal blindness in early infancy.^21^ Syndromic IRDs, though less common, constitute a more heterogeneous group comprising more than 80 described clinical entities. The most prevalent among them are ciliopathies, such as Usher syndrome (USH), Joubert syndrome, Bardet-Biedl syndrome (BBS), and Senior-Løken syndrome (SLS).^12^^,^^22^^,^^23^ Phenotypic variability among patients with the same IRD subtype can also be extensive and include differences in age of onset, rate of progression, severity, etc. Establishing a clinical diagnosis can therefore be a challenging task, often requiring a multidisciplinary approach that combines patient and family medical history with specialized diagnostic tests such as visual acuity and perimetry assessments, electroretinogram (ERG), fundus autofluorescence (FAF), and optical coherence tomography (OCT).^24^

Moreover, despite being monogenic conditions, IRDs are genetically highly heterogeneous and display multiple inheritance patterns (autosomal dominant [AD], autosomal recessive [AR], X-linked, and mitochondrial).^25^ Indeed, over 350 genes have been linked to retinal phenotypes, with syndromic forms accounting for ∼200 of them and RP alone being associated with ∼80 genes.^23^^,^^26^ Given this genetic complexity, next-generation sequencing (NGS) has become a cost- and time-effective method for the simultaneous screening of multiple genes, especially in large study cohorts. However, the current diagnostic rate, reported in the scientific literature, varies between 53% and 76%, based on results from multiple NGS techniques, such as panel sequencing, whole-exome sequencing (WES), and whole-genome sequencing (WGS).^27^^,^^28^^,^^29^^,^^30^^,^^31^^,^^32^^,^^33^ This diagnostic gap could be attributed to technical limitations, the existence of genes not yet linked to disease, or variants in regions not typically covered by targeted sequencing procedures, such as intronic or intergenic areas. However, emerging techniques, like *in vitro* RNA splicing assays^34^ and long-read sequencing,^35^ are being developed to address these challenges.

Another major hurdle in routine molecular diagnosis of IRDs is the use of incomplete or outdated lists of disease-associated genes,^36^ which hinders the proper design of real or virtual gene panels, an accurate interpretation of sequencing data, and the establishment of reliable genotype-phenotype associations. In this study, we aim to address this problem by providing an updated list of IRD-related genes, obtained from the latest scientific research, databases of human DNA variations, and repositories of gene expression data. This resource, curated by experts in the field, will be continually updated and made available on a dedicated website, ultimately to help researchers and clinicians identify disease-causing variants and support future discoveries and molecular diagnoses.

## Data mining and identification of genes associated with IRDs

**Figure 1:**
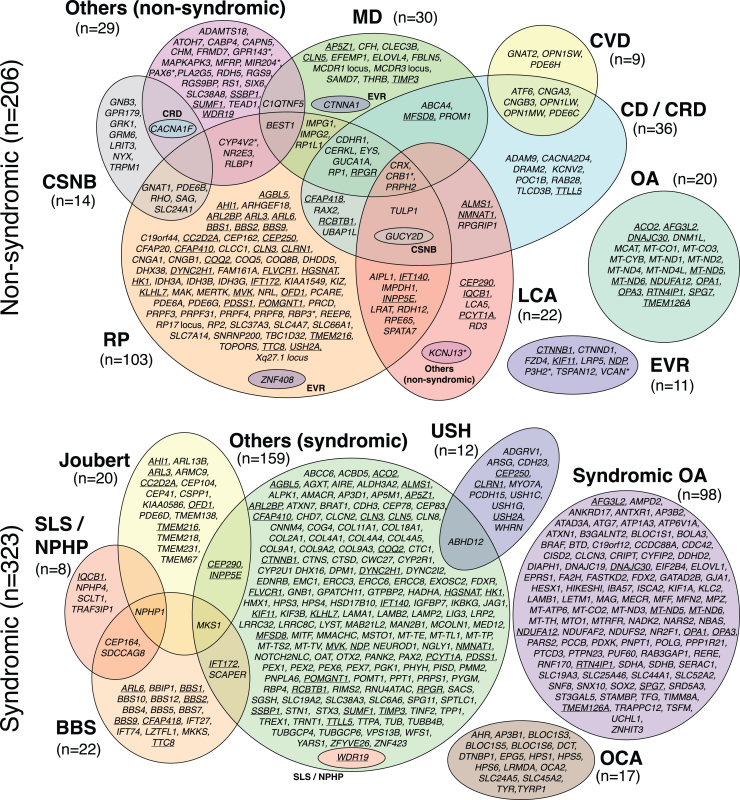
Venn diagram of genes and loci associated with IRDs (total = 470) Underlined genes are linked to both non-syndromic and syndromic phenotypes. Asterisks point to genes that can also be involved in non-retinal ocular diseases. *n*, number of genes.

Genes associated with IRDs were identified through data mining of public databases and published literature and were individually curated by at least two independent experts, according to the procedures described in the supplemental notes. At the end of the selection process, 470 genes (including four loci: *RP17* [MIM: 600852], *MCDR1* [MIM: 136550], *MCDR3* [MIM: 608850], and Xq27.1 [MIM: 301149]) were retained based on strong evidence of disease association (Figure 1). Another 196 genes were classified as “candidates,” primarily due to evidence from only a single affected family, and 17 genes were excluded due to insufficient evidence, conflicting data, or definitive proof of non-association with IRDs (Table S1).

## Phenotypes and inheritance

We chose a two-level approach to phenotype classification. The first level broadly distinguished between syndromic and non-syndromic phenotypes, based on the presence or absence of multisystemic signs in addition to retinal pathology. The second level defined 16 clinical subsets, including 14 specific groups (e.g., RP, MDs, etc.), and two heterogeneous categories that did not fit these groups: “other non-syndromic” and “other syndromic” (Table 1). Notably, we intentionally combined narrowly defined phenotypes such as Stargardt disease, choroideremia, or Sorsby fundus dystrophy into one of the 14 classes, as these entities are each associated with only one or very few genes (e.g., *ABCA4* [MIM: 601691], *CHM* [MIM: 300390], and *TIMP3* [MIM: 188826], respectively).

**Table 1:** Clinical classification of various inherited retinal diseases

**Phenotypes and abbreviations**	**Broad category**
Achromatopsia, color vision abnormalities, color blindness (CVD)	non-syndromic
Bardet-Biedl syndrome (BBS)	syndromic
Cone dystrophy, cone-rod dystrophy, Stargardt disease (CD/CRD)	non-syndromic
Congenital stationary night blindness (CSNB)	non-syndromic
Exudative vitreoretinopathy, Norrie disease (EVR)	non-syndromic
Joubert syndrome (Joubert)	syndromic
Leber congenital amaurosis (LCA)	non-syndromic
Macular dystrophy (MD)	non-syndromic
Oculocutaneous albinism, foveal hypoplasia (OCA)	syndromic
Optic atrophy, optic nerve hypoplasia (OA)	non-syndromic
Retinitis pigmentosa (RP)	non-syndromic
Senior-Løken syndrome, nephronophthisis (SLS/NPHP)	syndromic
Syndromic optic atrophy (syndromic OA)	syndromic
Usher syndrome (USH)	syndromic
Others non-syndromic	non-syndromic
Others syndromic	syndromic

**Figure 2:**
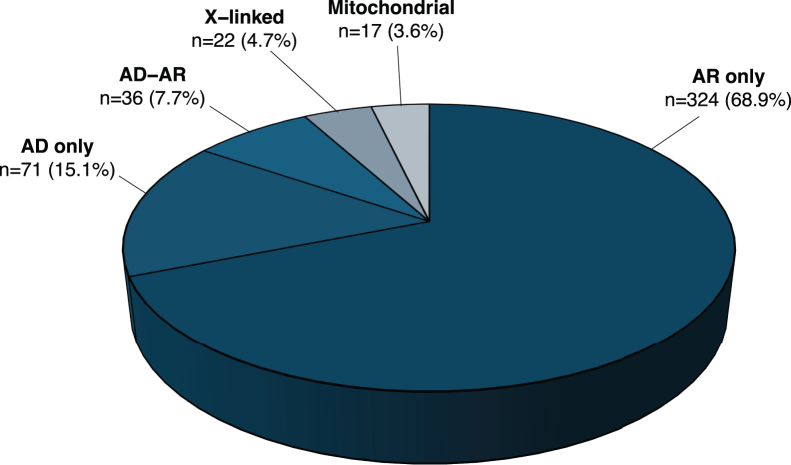
Inheritance mode of diseases associated with all curated genes and loci *n*, number of genes.

Out of the 470 curated genes and loci, 206 (202 genes and 4 loci) were associated with non-syndromic diseases in 9 phenotypic groups and 323 were associated with syndromic diseases in 7 phenotypic groups, with 59 genes found to be associated with both non-syndromic and syndromic diseases (Figure 1). Overall, most genes had variants responsible for AR inheritance (68.9%, *n* = 324), followed by AD (15.1%, *n* = 71), AD-AR (7.7%, *n* = 36), X-linked (4.7%, *n* = 22), and mitochondrial (3.6%, *n* = 17) heredity (Table S1; Figure 2).

Among the non-syndromic phenotypes, RP (50.0%, *n* = 103) involved the highest number of genes, followed by CDs/CRDs (17.5%, *n* = 36), MDs (14.6%, *n* = 30), LCA (10.7%, *n* = 22), OAs (9.7%, *n* = 20), CSNB (6.8%, *n* = 14), EVRs (5.3%, *n* = 11), and CVDs (4.4%, *n* = 9), while 29 (14.1%) genes were associated with other non-syndromic phenotypes (Figure 1). Notably, within these genes, 52 (25.2%) were associated with more than one non-syndromic phenotype (Table S1; Figure 1). For instance, *CRX* (MIM: 602225), *CRB1* (MIM: 604210), and *PRPH2* (MIM: 179605) were each linked to four phenotypes/clinical categories: RP, MDs, CDs/CRDs, and LCA; similarly, *GUCY2D* (MIM: 600179) was associated with RP, CDs/CRDs, LCA, and CSNB (Figures 1 and S1). In several instances, the genotype-phenotype relationship depended on the type of variant (e.g., loss of function [LoF] vs. missense] or on variant location within specific protein domains.^37^^,^^38^^,^^39^^,^^40^^,^^41^ For example, in *CRB1*, LoF variants are typically associated with LCA, while missense variants are more commonly linked to RP or MDs.^38^^,^^42^ Similarly, disease phenotypes associated with *GUCY2D* vary according to the type and location of the variant. Truncating mutations in the extracellular domain cause LCA, whereas missense changes in the protein kinase domain may result in LCA or CSNB, and variants in other parts of the protein are associated with RP, CSNB, CDs/CRDs, or LCA.^43^ Likewise, *RPGR* (MIM: 312610) can be linked to RP or CDs/CRDs, depending on the position of the variant along its primary sequence, in a gradient-dependent manner.^44^

For syndromic phenotypes, gene associations included syndromic OAs (30.3%, *n* = 98), BBS (6.8%, *n* = 22), Joubert syndrome (6.2%, *n* = 20), USH (3.7%, *n* = 12), SLS/nephronophthisis (NPHP; 2.5%, *n* = 8), oculocutaneous albinism (OCA)/foveal hypoplasia (5.3%, *n* = 17), and “others (syndromic)” (49.2%, *n* = 159), a broad and heterogeneous group involving additional systemic involvement beyond the eye (Figure 1).

Finally, 22 of the 59 genes associated with both syndromic and non-syndromic conditions encoded ciliary proteins, for which severe variants (typically LoF) tend to cause syndromic forms, while milder changes (typically missense or splicing variants) are more often associated with non-syndromic disease (e.g., in *ARL3* [MIM: 604695], *CEP290* [MIM: 610142], or *USH2A* [MIM: 608400]).^45^^,^^46^

## Historical perspective

**Figure 3:**
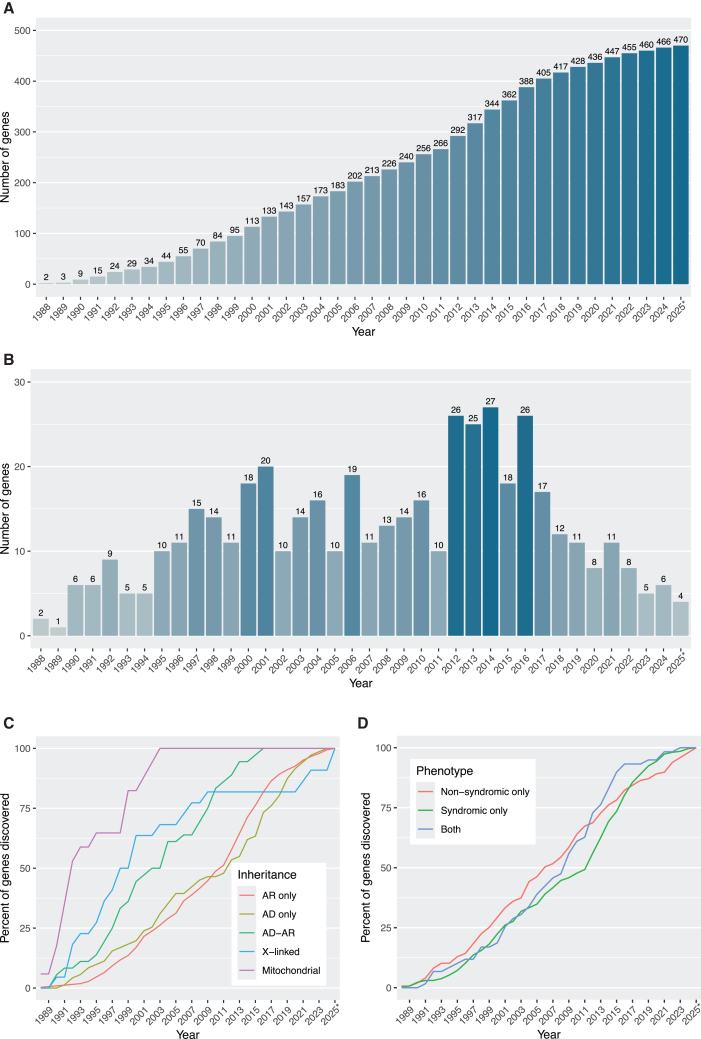
Discovery of IRD genes through time (A) Cumulative number of genes identified, per year. (B) Annual count of new gene discoveries. (C) Cumulative percentage of genes discovered, stratified by inheritance mode. (D) Cumulative percentage of genes discovered, stratified by broad phenotypic categories. ^∗^As of June 1, 2025.

Since the first identification of an IRD-associated gene in 1988 (*OAT* [MIM: 613349], linked to gyrate atrophy),^47^ the number of genes implicated in these conditions has increased steadily, with an average rate of ∼13 discoveries per year (Figure 3A). However, this growth has not been uniform. Until 2010, when gene identification primarily relied on linkage analysis, homozygosity mapping, or candidate gene approaches,^48^ the trend was essentially linear, with ∼11 new genes identified annually. In 2010, *FAM161A* (MIM: 613596) became the first IRD-associated gene to be identified using NGS,^49^ marking the beginning of a more rapid phase of gene discovery, which peaked at ∼26 genes per year and lasted until 2018. After 2018, however, the discovery rate declined to ∼8 genes per year, with only 6 genes identified in 2024, despite continued access to high-throughput sequencing. This slowdown may reflect the increasing rarity of newly identified genes in terms of gene-specific genetic prevalence, i.e., variants in these genes tend to account for a smaller number of affected individuals in the population (Figure 3B).

When stratifying IRD-associated genes by inheritance mode, we observed that those linked to phenotypes due to mitochondrial DNA defects were usually identified in the earliest years, followed by X-linked genes (Figure 3C), despite mitochondrial and X-linked forms representing the least common inheritance patterns in IRDs (Figure 2). This is likely due to the relative ease of detecting mitochondrial and X-linked inheritance, especially in large pedigrees. In addition, balanced X-autosome translocations in affected females and (micro)deletions in affected males facilitated the positional cloning of several genes on this chromosome (*CHM*, *NDP* [MIM: 300658], *RPGR*, and *RP2* [MIM: 300757]).^50^^,^^51^^,^^52^^,^^53^ Similarly, although AD inheritance accounts for only 15.1% of all curated genes (Figure 2), AD phenotypes also tended to be discovered earlier than AR phenotypes. This probably reflects the greater statistical power of linkage analysis in AD families compared to AR families of equivalent size (Figure 3C). In contrast, the rate of gene discovery was relatively similar over time between genes associated with non-syndromic and syndromic IRDs (Figure 3D).

## Functional classification

**Figure 4:**
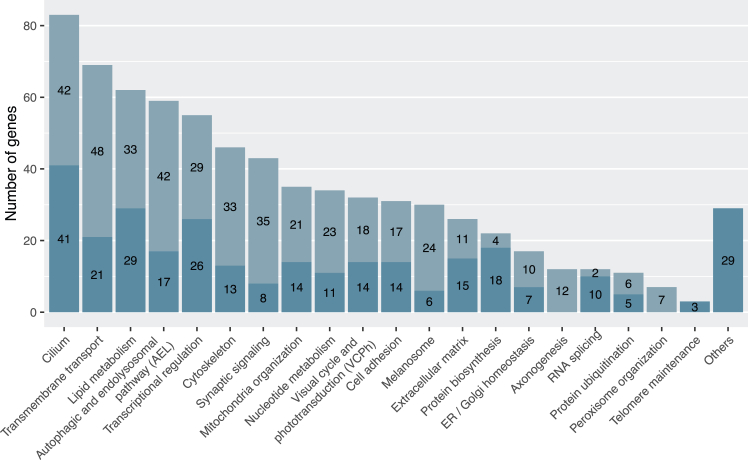
Functional categorization of IRD-associated genes The bar graph shows the number of genes assigned to each functional category relevant to IRDs. Bar segments indicate whether each gene is annotated in a unique category (blue) or appears in multiple categories (light blue). The "others" group includes genes with roles that could not be confidently assigned to the main categories.

We investigated the biological functions of the 466 curated genes (excluding the 4 loci) by stratifying them into 20 functional categories based on Gene Ontology (GO) terms, as detailed in the supplemental notes. Genes not assignable to any of these categories were manually reviewed and grouped under “others”. In total, 301 genes (64.6%) fell into a single functional category, while 165 genes (35.4%) were classified into multiple categories (Figure 4). These overlapping classifications enabled the identification of functionally related clusters.

For instance, “cilium” and “cytoskeleton” shared 20 genes, reflecting the cytoskeleton’s role as both a structural component of cilia and a regulator of ciliogenesis.^54^ Cilium also shared 8 genes with “melanosome,” and cytoskeleton shared 7 with “synaptic signaling,” due to the involvement of ciliary structures in melanosome transport and actin filaments in synaptic architecture.^55^^,^^56^^,^^57^^,^^58^^,^^59^ Similarly, “mitochondria organization” and “nucleotide metabolism” overlapped by 10 genes, as several rate-limiting steps of nucleotide metabolism occur in mitochondria.^60^ Each of these categories also shared 12–16 genes with “transmembrane transport,” related to mitochondrial electron transport processes.^61^ “Lipid metabolism” and “visual cycle and phototransduction” (VCPh) shared 9 genes through retinoid metabolism pathways.^62^ Furthermore, 10–11 “lipid metabolism” genes overlapped with “autophagic and endolysosomal pathway” (AEL) and “transmembrane transport.” Lastly, AEL, synaptic signaling, transmembrane transport, and melanosome shared 7–14 genes, presumably by virtue of their involvement in photoprotection, heterophagy, autophagy in the RPE, and synaptic signal transmission^63^^,^^64^ (Figure S2).

Cilium was the largest functional category, comprising 83 of the 466 genes (17.8%) (Figure 4). This was expected, given the critical role of cilia in photoreceptor physiology.^65^ The second and third largest categories were transmembrane transport (69 genes, 14.8%) and lipid metabolism (62 genes, 13.3%), encompassing proteins not only involved in VCPh but also in membrane-related metabolism that contributes to IRD pathogenesis. Notably, VCPh ranked tenth, representing only 32 genes (6.9%), reflecting a historical research shift: earlier gene identification efforts specifically targeted VCPh and retina-specific pathways, whereas modern studies are more unbiased.

We next evaluated the distribution of functional categories across three phenotype classes (syndromic, non-syndromic, both) and the 16 clinical subsets described before. In non-syndromic IRDs, the top categories were cilium (37 genes out of 202, 18.3%), transmembrane transport (32 genes, 15.8%), and VCPh (30 genes, 14.9%) (Figure S3A). VCPh was almost exclusively retina specific: 30 of its 32 genes (93.8%) caused non-syndromic IRDs, while other functional classes showed broader phenotypic associations, with less than 25% of their genes confined to non-syndromic cases.

Cilium and VCPh genes were most commonly associated with RP, while transmembrane transport genes were linked equally to RP and OA (Figure S3B). OA-associated genes also belonged to mitochondria and nucleotide metabolism categories, which primarily contribute to OA. CSNB was mainly associated with synaptic signaling, and VCPh genes were consistent with defects in the phototransduction cascade and ribbon synapses. EVR was predominantly linked to “extracellular matrix,” “cell adhesion,” and “transcriptional regulation” genes. Although VCPh genes represented only 30 of the 202 entries associated with non-syndromic IRDs, they were associated with the broadest range of clinical subtypes—including LCA, MDs, CVDs, and CRDs (Figure S3B).

In syndromic IRDs, cilium dominated again (69 genes, 21.4%), reflecting the multisystem involvement typical of ciliopathies (Figure S3A). USH, Joubert syndrome, SLS, and BBS were mainly linked to cilium and cytoskeleton categories (Figure S3C). OCA was represented mainly by melanosome genes, highlighting the dual role of melanin pathways in ocular and cutaneous pigmentation.^66^ Syndromic OA was associated with nearly all categories, consistent with its complex etiology. In contrast, as mentioned, VCPh was underrepresented in syndromic IRDs, with only two implicated genes (0.6%), both involved in multiple pathways: *GNB1*, which causes a neurological phenotype,^67^ and *RBP4*, associated with skin involvement^68^ (Figure S3C).

## Inheritance of disease and variant classes

**Figure 5:**
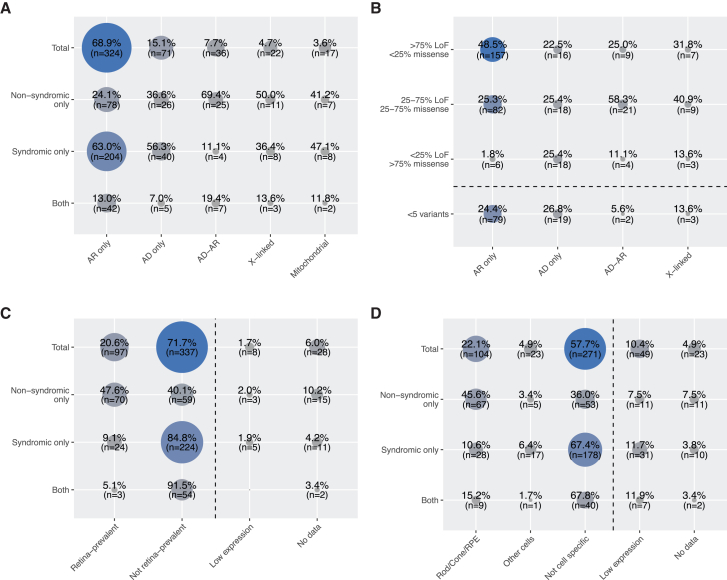
Inheritance and expression features of IRD genes Co-occurrence matrices between (A) inheritance mode and broad phenotypic categories, (B) inheritance mode and type of pathogenic variants, (C) expression in retina/other tissues and broad phenotypic categories, and (D) expression in retinal cell types and broad phenotypic categories. *n*, number of genes.

Details about inheritance and phenotypes are shown in Figures 2 and 5A. The majority of curated genes were associated with an AR inheritance pattern across syndromic and non-syndromic phenotypes. AR-associated genes were more frequently linked to purely syndromic presentations (63.0%, *n* = 204) than to non-syndromic ones (24.1%, *n* = 78). AD inheritance was slightly more common in genes associated with syndromic conditions (56.3%, *n* = 40) than with non-syndromic ones (36.6%, *n* = 26), though the difference was modest, and AD inheritance was not observed for prevalent conditions such as USH, Joubert syndrome, BBS, etc. (Figure S4). In contrast, the discrepancy was more pronounced among genes associated with both AD and AR inheritance, with 25 (69.4%) linked to non-syndromic diseases vs. only 4 (11.1%) associated with syndromic conditions. X-linked IRDs were evenly represented across syndromic and non-syndromic forms, as well as specific clinical subtypes. Mitochondrial inheritance was observed in both phenotype classes but was almost exclusively associated with OA. This condition results from the degeneration of RGCs, which transmit visual signals to the brain via the optic nerve and rely on high levels of ATP, a process critically dependent on intact mitochondrial function.^19^ Details on the inheritance of syndromic vs. non-syndromic phenotypes are shown in Figure S4.

Next, we investigated whether the spectrum of pathogenic and likely pathogenic (PLP) variants correlates with inheritance mode or phenotypes. Each IRD-associated gene was assigned to one of four categories, based on ClinVar^69^ data: (1) > 75% LoF variants, (2) 25%–75% LoF and missense variants (mixed), (3) > 75% missense variants, or (4) fewer than 5 PLP variants (rare). We observed a strong correlation between types of variants and inheritance patterns. Genes associated with AR phenotypes were mostly enriched for LoF variants, followed by mixed or rare classes (Figure 5B). This is consistent with the haplosufficiency of most recessive alleles and the fact that AR conditions typically result from complete protein loss. By contrast, AD conditions generally result from heterozygous gain-of-function, dominant-negative, or haploinsufficient mutations. Accordingly, genes in the AD group were nearly evenly distributed across all four variant categories.^70^ Likewise, genes with AD-AR or X-linked inheritance, neither strictly dominant nor recessive, were most commonly found in the mixed LoF/missense group (Figure 5B). LoF variants were also the most prevalent type of DNA changes overall and mostly associated with syndromic conditions and ubiquitously expressed genes (Figure S5).

## Gene expression

To investigate a potential correlation between tissue-specific gene expression and the syndromic or non-syndromic nature of retinal disease, we analyzed bulk RNA sequencing (RNA-seq) data from various tissues using the FANTOM5 dataset.^71^ Based on gene expression levels in retinal vs. non-retinal tissues, we defined four categories: “retina-prevalent” for genes with significantly higher expression in the retina compared to other tissues, “not retina-prevalent” for genes with expression in the retina but also in other tissues, and “low retinal expression” or “no data” for genes with very low expression in the retina or that were absent from the FANTOM5 dataset, respectively (see supplemental notes).

We found that 47.6% (*n* = 70) of genes linked only to non-syndromic IRDs were classified as retina prevalent, which aligns well with classical mechanisms of pathogenesis. Conversely, 40.1% (*n* = 59) of genes associated with diseases restricted to the retina were ubiquitously expressed across tissues (Figure 5C). This is not surprising, as many housekeeping genes, essential for all cells, have been previously implicated in non-syndromic IRDs. These include splicing factor genes and genes involved in core metabolic pathways such as the TCA cycle, coenzyme Q biosynthesis, and nucleotide metabolism.^72^^,^^73^^,^^74^^,^^75^ A widely accepted hypothesis for this paradox is the retina’s intrinsic sensitivity to even minimal metabolic or functional disturbances, making it particularly vulnerable compared to other tissues or organs. Supporting this, the majority of genes implicated in syndromic IRDs (84.8%, *n* = 224) or involved in both syndromic and non-syndromic forms (91.5%, *n* = 54) were “not retina-prevalent,” many of which are linked to ciliopathies (Figure 5C).

We also examined cell-specific gene expression within the retina in relation to disease phenotype. Using single-cell RNA-seq (scRNA-seq) data, we first classified IRD-associated genes into five categories: “rod/cone/RPE,” “other cells,” “not cell specific,” “low expression,” and “no data” (Figure 5D). Notably, 45.6% (*n* = 67) of genes linked to non-syndromic IRDs showed specific expression in photoreceptors or the RPE, compared to 36.0% (*n* = 53) falling into the “not-cell-specific” group. This supports the established concept that non-syndromic IRDs often result from dysfunction or degeneration of cones, rods, RPE cells, or combinations thereof.^76^ This observation is reinforced by the fact that most genes linked to syndromic IRDs (67.4%, *n* = 178) showed broad expression across multiple retinal cell types, reflecting their functional relevance in other organs as well. Interestingly, over 15.3% of IRD genes had either minimal or undetectable expression in retinal cells. In addition to developmental stage-specific expression, this is likely due to technical limitations of scRNA-seq, particularly transcript dropout events, rather than true biological absence.^77^

**Figure 6:**
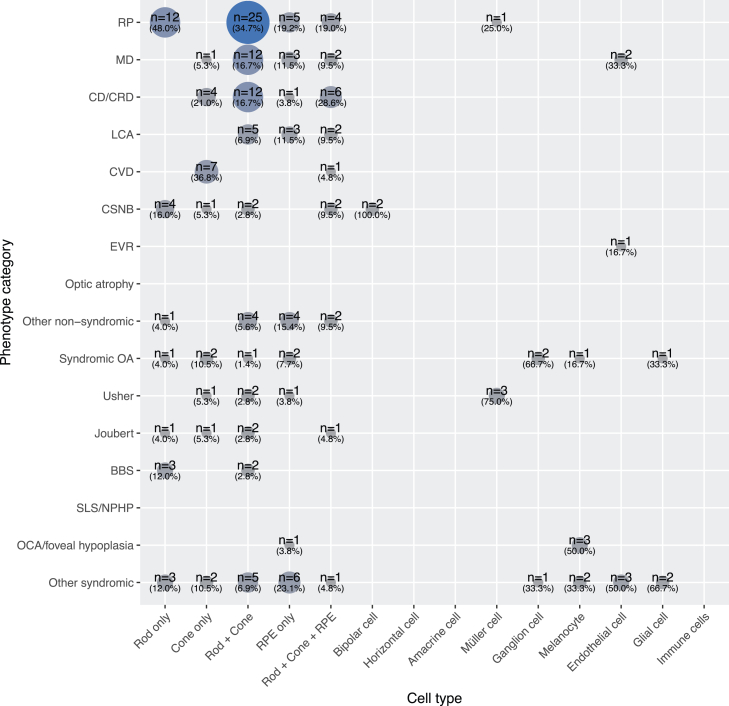
Co-occurrence matrix between phenotypes and retinal single-cell gene expression data *n*, number of genes.

We then analyzed scRNA-seq expression patterns in the context of specific clinical phenotypes (Figures 6 and S6). As expected from clinical and electrophysiological studies, RP was linked to genes expressed in photoreceptors and RPE cells. The involvement of rod-specific genes matches the typical clinical course: initial night blindness followed by progressive rod and cone degeneration, culminating in tunnel vision. Macular dystrophies and CDs or CRDs could not be reliably distinguished based on scRNA-seq alone, likely due to overlapping expression profiles in the same cell types and differences primarily in the affected retinal regions (macula vs. the entire retina). Interestingly, CDs/CRDs showed a strong involvement of genes expressed exclusively in cones, consistent with cone-only disease mechanisms. LCA, a condition characterized by early-onset, severe vision loss, involved genes affecting rods, cones, and/or the RPE (but not restricted to a single photoreceptor type).^78^ This suggests that LCA results from disruptions to fundamental cellular processes necessary for the function of all photoreceptors and the RPE. In CVDs, gene expression was limited to cones, which is consistent with the fact that color vision depends entirely on this photoreceptor subtype. CSNB is associated with nyctalopia from birth and is typically caused by mutations in genes involved in synaptic junctions between photoreceptors (primarily rods) and bipolar cells, although some CSNB subtypes may also involve milder cone dysfunction, as indicated by our analysis (Figures 6 and S6).

The scRNA-seq expression patterns for syndromic IRDs were more diverse. For instance, genes implicated in OCA and foveal hypoplasia were mostly expressed in melanocytes, consistent with their role in melanin biosynthesis. Conversely, some genes associated with syndromic OA were expressed in melanocytes, retinal ganglion, and glial cells, while three USH genes showed expression in Müller cells. Notably, *USH1G* (MIM: 607696), *CLRN1* (MIM: 606397), and *CDH23* (MIM: 605516), linked to USH, have been shown to be involved in the disease through Müller cell dysfunction as well.^79^^,^^80^^,^^81^ Finally, the group of other syndromic phenotypes involved genes expressed in a wide range of retinal cell types. This broad expression likely reflects real biological relevance rather than an artifact of phenotype grouping. In contrast, genes associated with non-syndromic conditions (although more numerous overall) tended to show restricted expression limited to photoreceptors and the RPE (Figure 6, top).

## Comparison with existing resources and diagnostic relevance

An important strength of RetiGene is its active refinement of content to improve clinical diagnostic accuracy. Compared to existing databases, RetiGene includes a greater number of IRD-associated genes, particularly those linked to syndromic forms, as well as a relatively high proportion of candidate genes (Table S2). These differences likely reflect the effects of manual expert curation combined with stricter inclusion criteria (see supplemental notes).

Such criteria also enable the systematic exclusion of genes with strong contradictory evidence, which is particularly relevant in diagnostic workflows. For example, *UNC119* is still present in many IRD gene panels, despite compelling evidence that its association with dominant disease is likely incorrect. This includes the high frequency of reported pathogenic variants in the general population, lack of co-segregation with disease, and the gene’s tolerance to truncating variants.^82^^,^^83^^,^^84^^,^^85^^,^^86^ Using its standardized curation framework, RetiGene has classified *UNC119* as “discarded,” providing a clear signal to diagnostic laboratories that variants in this gene should not be considered clinically meaningful.

## Implementation of the database

All the data presented here are hosted on a website with an intuitive user interface, ensuring easy navigation and accessibility (https://retigene.erdc.info/). The content will be regularly updated through literature search and user requests to reflect the latest information, maintaining both accuracy and relevance for users.

## Conclusions

In summary, we have assembled a freely accessible online database of genes involved in IRDs, which will be continuously updated. This comprehensive catalog is intended to support the identification of disease-causing variants, the development of more accurate genetic testing panels, and a deeper understanding of the molecular mechanisms underlying these conditions. We believe that this resource will contribute to the development of new targeted therapies, improve diagnostic precision, and ultimately enhance patient care for individuals affected by IRDs.

## Data and code availability

All curated genes and figures from this study are available on the RetiGene website (https://retigene.erdc.info/).

## Acknowledgments

The authors would like to acknowledge the following funding bodies. E.D. was supported by the 10.13039/501100004385Ghent University Special Research Fund (BOF22/DOC/229). M.B. and L.V. were supported by the 10.13039/501100003130Research Foundation Flanders (1SD8924N to M.B. and 11PS324N to L.V.). L.F.-C. was supported by the Centro de Investigación Biomédica en Red (CIBER). G.G.G. was supported by the 10.13039/501100004587Instituto de Salud Carlos III (ISCIII) (CP22/00028 and PI22/01371) and the 10.13039/501100000780European Union, through the HORIZON programme (HORIZON-HLTH-2023-TOOL-05-04, BETTER, 101136262). L.K.H. was supported by the 10.13039/501100000262Foundation Fighting Blindness Project Program Award (PPA-0622-0841-UCL). C.A. was supported by ISCIII of the Spanish Ministry of Health (PI22/00321), Centro de Investigación Biomédica en Red Enfermedades Raras (CIBERER, 06/07/0036), IIS-FJD BioBank (PT13/0010/0012), the Organización Nacional de Ciegos Españoles (ONCE), the 10.13039/501100008530European Regional Development Fund (FEDER), and the University Chair UAM-IIS-FJD of Genomic Medicine. S.B. was supported by 10.13039/501100002426Fondazione Telethon (PE00000006 and CUP H93C22000660006-MNESYS). F.C. was supported by the Research Foundation Flanders (G0ACQ24N). F.P.M.C. was supported by the 10.13039/100001116Foundation Fighting Blindness USA (BR-GE-0120-0775-LUMC). C.F.I. and C.T. were supported by the RP Fighting Blindness and Fight for Sight UK (RP Genome Project GR586). R.K.K. was supported by the 10.13039/501100020867Montreal Children's Hospital Foundation, the Vision Sciences Research Network (VSRN), the 10.13039/100000002National Institutes of Health (R01 EY030499-01, Dr. Lentz), the 10.13039/501100000024Canadian Institutes of Health Research (CIHR), 10.13039/501100000262Fighting Blindness Canada (FBC), and 10.13039/501100000156Fonds de Recherche du Québec - Santé (FRQS). R.K.K. also participates in the NAC Attack clinical trial, which is funded by the National Institutes of Health via grants UG1EY033286, UG1EY033293, UG1EY033286, and UG1EY033292. J.M.M. is supported by ISCIII (PI22/00213, AC21_2/00022, and FORT23/00021, the latter co-funded by the European Union) and by the 10.13039/501100003359Generalitat Valenciana (CIPROM/2023/26). C.R. is supported by the 10.13039/501100001711Swiss National Science Foundation (grant no. 204285).

## Declaration of interests

The authors declare no competing interests.

## Web resources

OMIM, https://www.omim.org
ClinVar, https://www.ncbi.nlm.nih.gov/clinvar/
RetiGene, https://retigene.erdc.info/
RetNet, https://retnet.org/
Genomics England PanelApp Retinal disorders, https://panelapp.genomicsengland.co.uk/panels/307/
CeGaT Eye Diseases panel, https://cegat.com/diagnostics/rare-diseases/eye-diseases
LOVD, https://www.lovd.nl/
ClinGen Retina GCEP, https://clinicalgenome.org/affiliation/40072/

## Supplemental information

Document S1. Figures S1–S6 and supplemental notes

Table S1. List of all curated genes with their corresponding annotations

Table S2. Comparison of RetiGene with other curated retinal databases and panels

Table S3. Terms used for OMIM and PubMed data mining

Table S4. List of functional categories, corresponding GO terms, and PMIDs of relevant literature for genes that underwent manual classification

Document S2. Article plus supplemental information
