# Supplementary material for: RetiGene, a comprehensive gene atlas for inherited retinal diseases
Source: Am J Hum Genet. 2025 Sep 16;112(10):2253–65. doi: 10.1016/j.ajhg.2025.08.017 (PMC12696501; doi:10.1016/j.ajhg.2025.08.017)
Supplement: Document S1. Figures S1–S6 and supplemental notes [file mmc1.pdf]

The American Journal of Human Genetics, Volume *112*

Supplemental information

RetiGene, a comprehensive gene atlas for inherited retinal diseases

Carlo Rivolta, Elifnaz Celik, Dhryata Kamdar, Francesca Cancellieri, Karolina Kaminska, Mukhtar Ullah, Pilar Barberán-Martínez, Manon Bouckaert, Marta Cortón, Emma Delanote, Lidia Fernández-Caballero, Gema García García, Lara K. Holtes, Marianthi Karali, Irma Lopez, Virginie G. Peter, Nina Schneider, Lieselot Vincke, Carmen Ayuso, Sandro Banfi, Beatrice Bocquet, Frauke Coppieters, Frans P.M. Cremers, Chris F. Inglehearn, Takeshi Iwata, Vasiliki Kalatzis, Robert K. Koenekoop, José M. Millán, Dror Sharon, Carmel Toomes, and Mathieu Quinodoz

Supplemental Notes

Operational definition

In this study, we define IRDs as conditions that affect the retina directly, such as diseases caused by mutations in *RHO* (MIM: 180380) leading to rod photoreceptor death,[1] *CNGA3* leading to non-functional cones,[2] or *OPA1* (MIM: 605290) leading to ganglion cell death.[3] We also consider conditions that involve the retina secondarily due to pathology originating elsewhere, such as mutations in *FZD4* (MIM: 604579), which cause retinal detachment as a consequence of abnormal retinal vascularization,[4] or in *ABCC6* (MIM: 603234), which is mainly expressed in the liver and leads to abnormal calcium accumulation in Bruch's membrane and its subsequent breakage.[5]

Gene curation

The initial list of genes associated with IRDs was compiled based on data from publicly available sources and then manually curated. The first source was the ClinGen (Retina GCEP, accessed on December 31st, 2024),[6], comprising 119 entries. The second source was RetNet (accessed on January 16, 2025),[7] from which we extracted the full annotated list of IRD genes and loci, totaling 353 entries. The third source was the Genomics England PanelApp (Version 7 of the "Retinal Disorders" panel),[8] which included 430 genes and loci. The fourth source was a IRD-specific list of the Leiden Open Variation Database (LOVD), curated and kindly provided by Prof. Frans Cremers. The fifth source was the CeGaT Eye Disease Panel, including 232 genes as of July 10, 2025. The sixth source was the Online Mendelian Inheritance in Man (OMIM) database (accessed on January 16, 2025),[9] which was queried using the phenotype-specific keywords listed in Table S3, as well as other filters, including: "Gene Map Locus", "Clinical Synopsis", and OMIM's term-specific symbols star (*, genes with known sequence), plus (+, gene with known sequence and phenotype), and dash (−, phenotype with known molecular basis). The combined filtered output initially yielded 1047 entries, from which 609 unique genes were retained after deduplication. The seventh and final source was a structured

literature review using PubMed (accessed on June 1st, 2025), which was also queried with the terms listed in Table S3. To refine search results toward gene-relevant findings, we included Boolean keywords such as “mutation”, “variant”, or “genotype” restricted to the Title or Abstract. All queries were manually reviewed, and a subset of newly associated genes (including *GPATCH11*, *AP5M1*, *AP5Z1*, and *COQ8B*, etc.) was added to the initial list. To ensure completeness, we additionally consulted key review articles.[10-14]

After gathering data from all sources, the gene entries were merged into a master dataset. Following deduplication, the unified list contained 683 unique genes and loci. As of June 1st, 2025, data collection was concluded.

At the end of the collection procedure, each gene was independently evaluated by two experts and included in the downstream analysis if it met either of the following criteria: (i) it harbored distinct pathogenic variants in two or more unrelated individuals or families showing consistent disease phenotype and inheritance pattern; or (ii) it carried the same variant in at least two unrelated individuals or families, supported by strong functional evidence of pathogenicity. Genes that did not fulfill these criteria were classified as “Candidates.” Genes for which conflicting evidence or definitive proof of non-association with IRDs existed were excluded. Loci identified from linkage and association studies without known causative variant(s) were excluded. If they had known causative variants, then they were checked for the criteria of inclusion as stated above. For example, RP17 is a known locus associated with AD-RP due to the presence of complex structural variants which result in ectopic expression of *GDPD1* (MIM: 616317).[15] The RP17 locus was therefore retained since these variants segregated in more than 20 families, thus meeting our criteria of inclusion (i).

Historical perspective selection criteria

Curated genes were annotated with the year of the publication that first linked pathogenic variant(s) in these genes with any form of IRDs.

Functional classification of genes

The 466 curated genes were annotated for biological process GO terms (GOTERM_BP_FAT) using the Functional Annotation tool from the Database for Annotation, Visualization, and Integrated Discovery (DAVID) knowledgebase (version 2021).[16] A total of 446 genes were annotated with over 2500 GO terms in total. Based on these annotations, we grouped the genes into 20 functional categories, as listed in Table S4. Lastly, genes that lacked GO term annotations that fit into these 20 categories were individually assessed by literature review and either manually assigned to one or more of them or grouped into the category "Others". Corresponding PubMed IDs (PMIDs) for these manual annotations are also provided in Table S4.

Manual annotation was also performed for genes that required refinement beyond the DAVID output due to an unspecific GO term assigned. For example, *PDE6B* (MIM: 180072) and *PDE6C* (MIM: 600827) were directly grouped into "visual cycle and phototransduction" functional category based on the GO terms listed in Table S4, while other related genes, such as *PDE6A* (MIM: 180071)*, PDE6G* (MIM: 180073)*, and PDE6H* (MIM: 601190), were annotated under the more generic term "visual perception" (GO:0007601), necessitating their reclassification for consistency across functionally similar genes.

Tissue and retinal cell type expression specificity

To investigate tissue expression of the 470 curated genes and loci in the human transcriptome, the FANTOM5 RNA expression dataset[17] was downloaded from The Human Protein Atlas.[18] The dataset contains normalized transcript-per-million (nTPM) values for 18,287 genes in 60 different human tissue samples, including the retina. The average nTPM values of genes were calculated by grouping some of the tissues as follows: "brain_max" = amygdala, caudate, cerebellum, thalamus, hippocampus, nucleus accumbens, temporal cortex, pituitary gland, putamen, postcentral gyrus, spinal cord, substantia nigra, corpus callosum, frontal lobe, insular cortex, olfactory bulb, pons, occipital pole, occipital lobe, occipital cortex, medulla

oblongata, medial temporal gyrus, medial frontal gyrus; “glands_max” = salivary gland, thyroid gland, thymus; “digestive_max” = colon, esophagus, small intestine, appendix, gallbladder, smooth muscle; “heart” = heart muscle; “liver”; “lung”; “pancreas”; “muscle” = skeletal muscle; “lymph_node” = lymph node; “diversive_max” = prostate, spleen, tongue, urinary bladder, adipose tissue, breast, cervix, endometrium, ovary, vagina, placenta, seminal vesicle. Values from testis, kidney, and retina were not grouped. At the end of this process, tissue samples were organized into 13 distinct sets for downstream analysis.

Then for each gene, the z-score$_{retina}$ (nTPM$_{retina}$ vs nTPM of other tissues) and the expression ratio (ratio$_{retina}$, nTPM$_{retina}$ / nTPM$_{max}$ in other tissues) were calculated using a custom R script. Based on these values, genes were further categorized as follows: “Retina prevalent” = z-score$_{retina}$ > 3 AND ratio$_{retina}$ > 3 AND nTPM$_{retina}$ > 1; “Not retina prevalent” = (z-score ≤ 3 OR ratio$_{retina}$ ≤ 3) AND nTPM$_{retina}$ > 1; “Low expression” = nTPM$_{retina}$ ≤ 1; “No data” = genes that are not included in the FANTOM5 dataset.

Similarly, for the investigation of the single-cell expression of the 470 curated genes within the human retinal tissue, library-normalized transcripts per cell of the adult human peripheral retina were downloaded from a public repository.[19] This dataset contains expression normalized to 10,000 transcript counts per cell type for 57118 genes and 53 cell/cell subtypes of the retina. The 53 cell and cell subtypes were condensed into 19 major groups (Rods, Cones, RPE, Horizontal cells, Amacrine cells, Bipolar Cells, Ganglion cells, Muller cells, Astrocytes, Glial cells, Choroidal melanocyte, Microglial, Monocytes, NK cells, T cells, Mast cells, Pericytes, Fibroblasts, Vascular endothelial cells) by taking the average expression of the cell subtypes. For example, group ‘Cones’ is the average of the L/M and S cone sub-cell types. Further, 5 broader groups were created from these 19 major groups namely, “Rods+Cones” (average of the major groups Rods and Cones), “Rods+Cones+RPE” (average of the major groups Rods, Cones and RPE), “Endothelial cell” (average of major groups Pericytes, Fibroblasts, and Vascular endothelial cells), “Immune cells” (average of major

groups NK cells, T cells, and Mast cells), and “Glial cells” (average of major groups Astrocytes, glial cells and microglial cells).

Then, for each gene, the z-score (normalized expression count of each group vs average of the normalized expression count of remaining groups) and the expression ratio (normalized expression count of each group divided by the average of the normalized expression count of remaining groups) were calculated on a custom R script. Genes were said to be specific to one of the 24 categories (19 major groups and 5 broader groups) based on z-score, ratio, and nTPM as follows: “Group specific” = $\text{z-score}_{group} > 3$ AND $\text{ratio}_{group} > 3$ AND $\text{nTPM}_{group} > 1$;if they did not meet this criteria for any of the 24 categories, they were marked “Not cell specific”; “Low expression” = $\text{nTPM}_{group} \leq 1$; “No data” = genes that are not included in the scRNAseq dataset. For interpretability, we collapsed the results into three overarching specificity categories: “Rods/Cones/RPE” if the gene passed the “Group specific” threshold to be specific to either rods, cones, or RPE, “other cell” if the gene passed the threshold to be specific to either horizontal cells, amacrine cells, bipolar cells, ganglion cell, muller cell, melanocyte, endothelial cell, glial cell or immune cell and “None” if it was found not to be specific to any of the retinal cell groups.

Variant classification

VCF file containing variant information for all the curated genes was downloaded from the ClinVar database (version of 21.01.2023). PLP variants were selected and annotated using ANNOVAR.[20] Then, missense and LoF variants were counted per gene. LoF mutations were defined if the variant led to a canonical splicing event, stopgain mutation, or insertion/deletion leading to a frameshift.

Website maintenance and updates

The results obtained in this work and, specifically, the list of IRD-associated genes, has been made publicly available on a dedicated website (retigene.erdc.info).

Every month, the gene list and the website content will be updated through searches in OMIM and PubMed, as described above. A time filter will be added to review updates from June 1st 2025. Following this search, all genes and articles identified will be manually curated and potentially incorporated into the website. Importantly, we would welcome any feedback from other researchers / users who may directly suggest additions or amendments to the gene list and the website (e.g., about gene associations they have recently discovered or published).

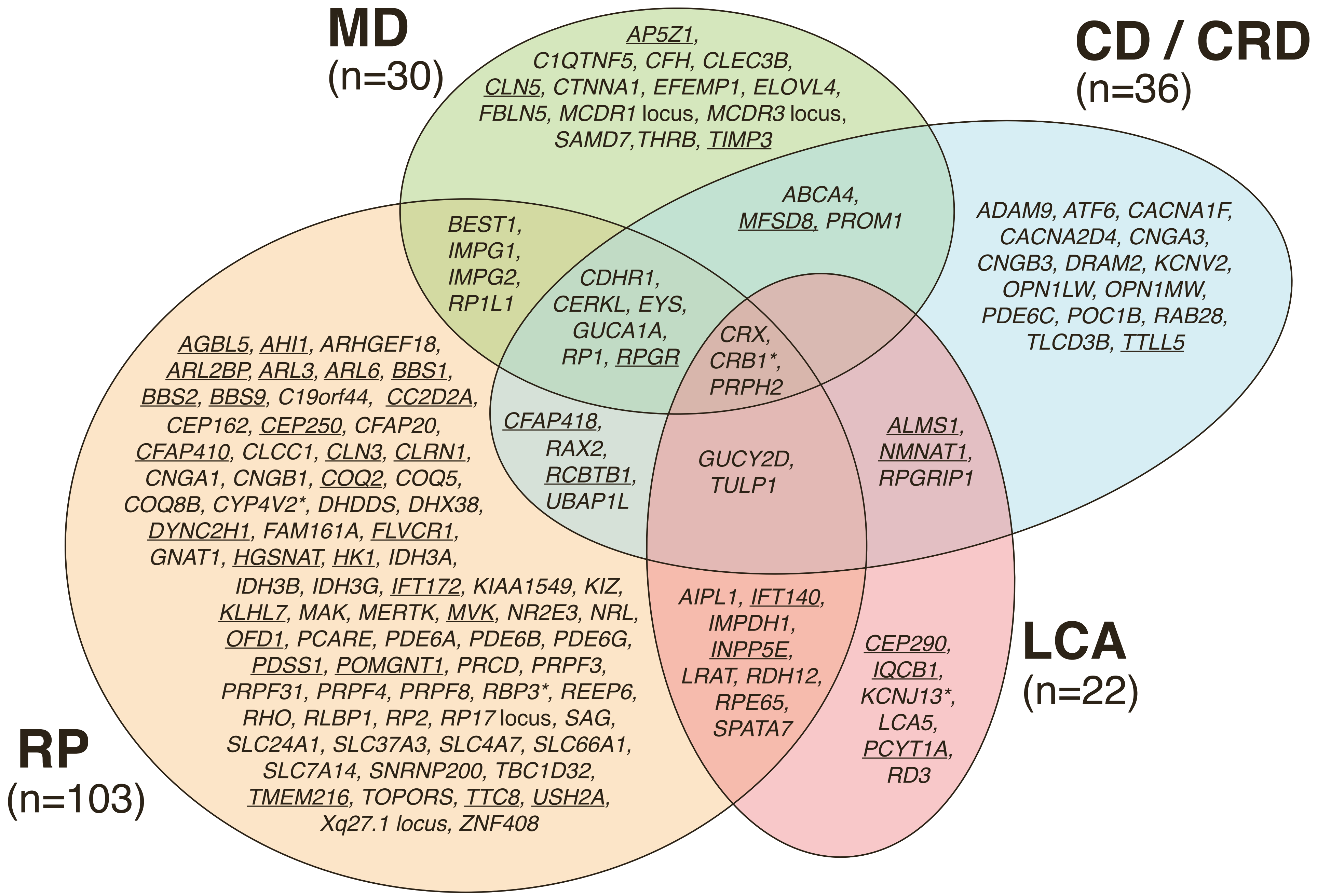

Figure S1: Venn diagram of genes and loci associated with the most common non-syndromic IRDs. Underlined genes are linked to both non-syndromic and syndromic phenotypes. Asterisks point to genes that can also be involved in non-retinal ocular diseases.

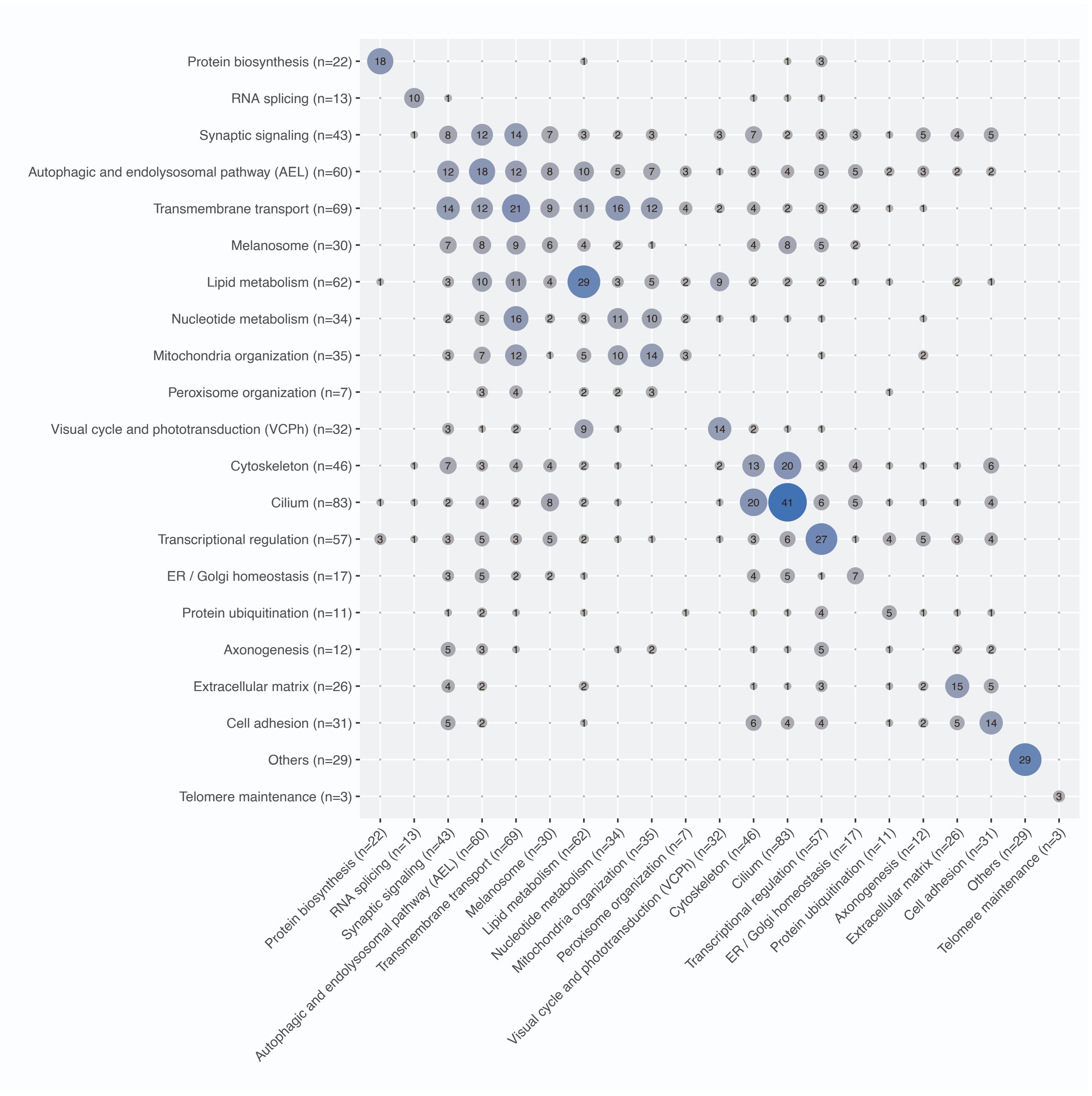

Figure S2. Overlap among functional categories associated with IRD genes. Functional categories are listed along both axes. n (or plain numbers), number of genes.

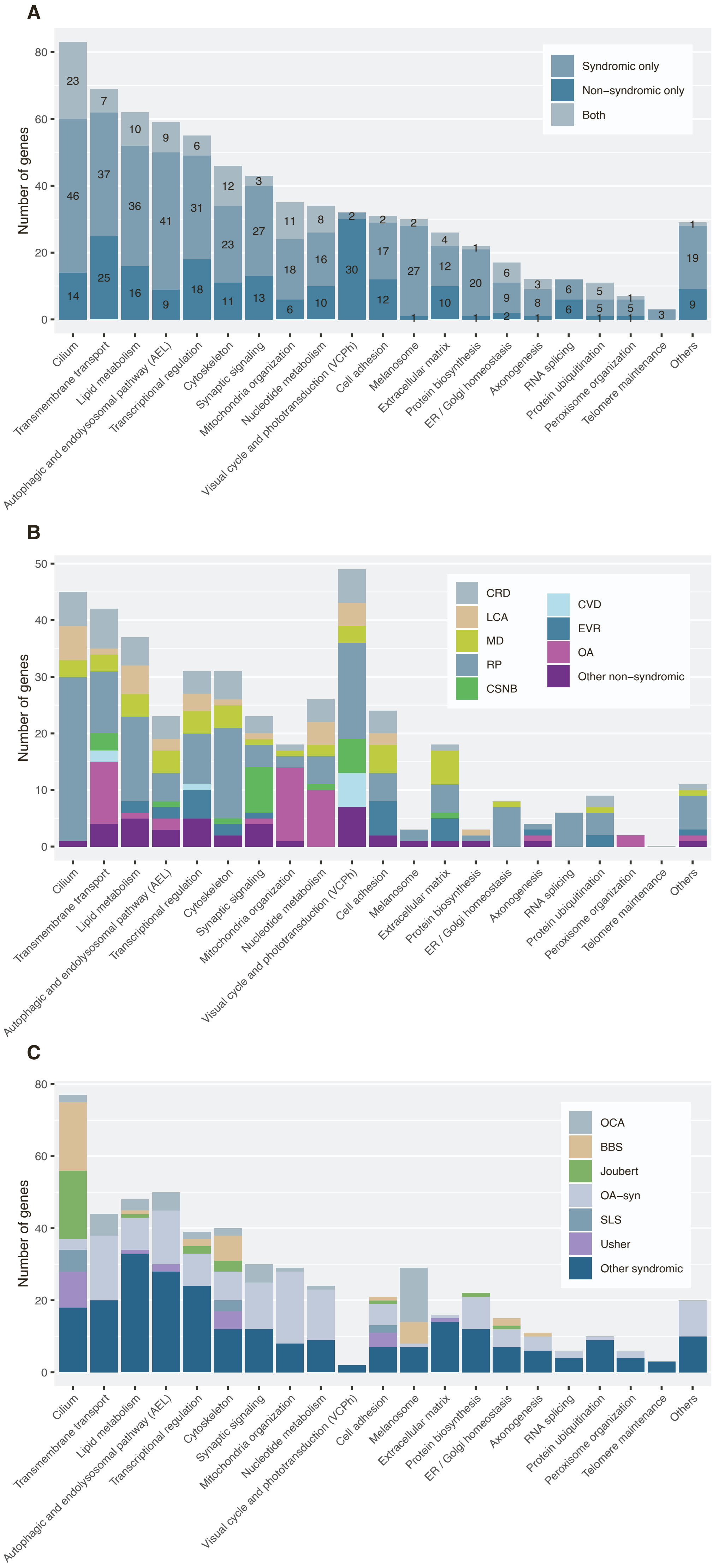

Figure S3: Functional classification of genes, stratified by phenotypes. (A) Broad phenotypic categories. (B) Non-syndromic phenotypes. (C) Syndromic phenotypes.

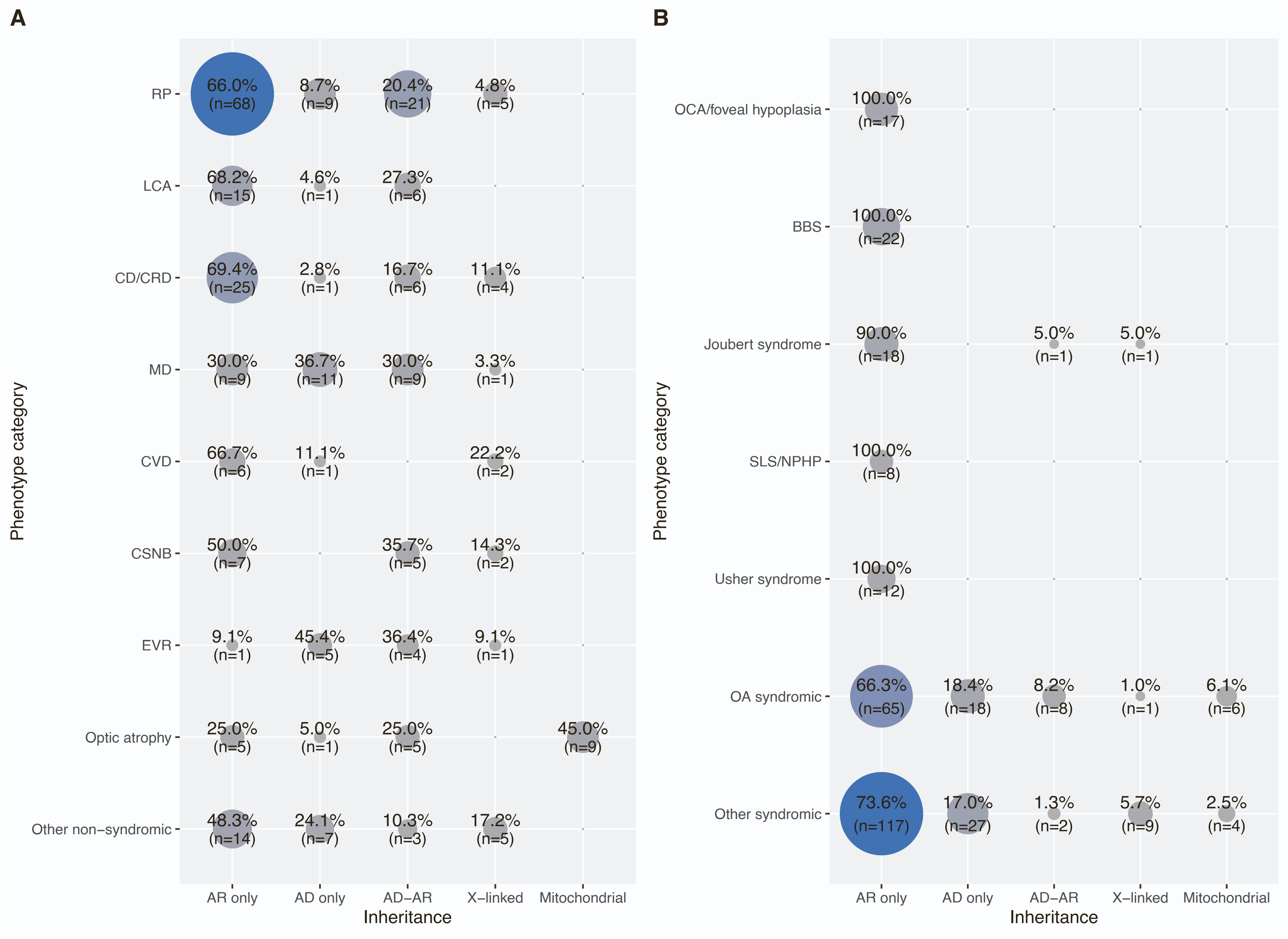

Figure S4: Co-occurrence matrix between phenotypes and their inheritance. (A) Non-syndromic phenotypes. (B) Syndromic phenotypes. n, number of genes.

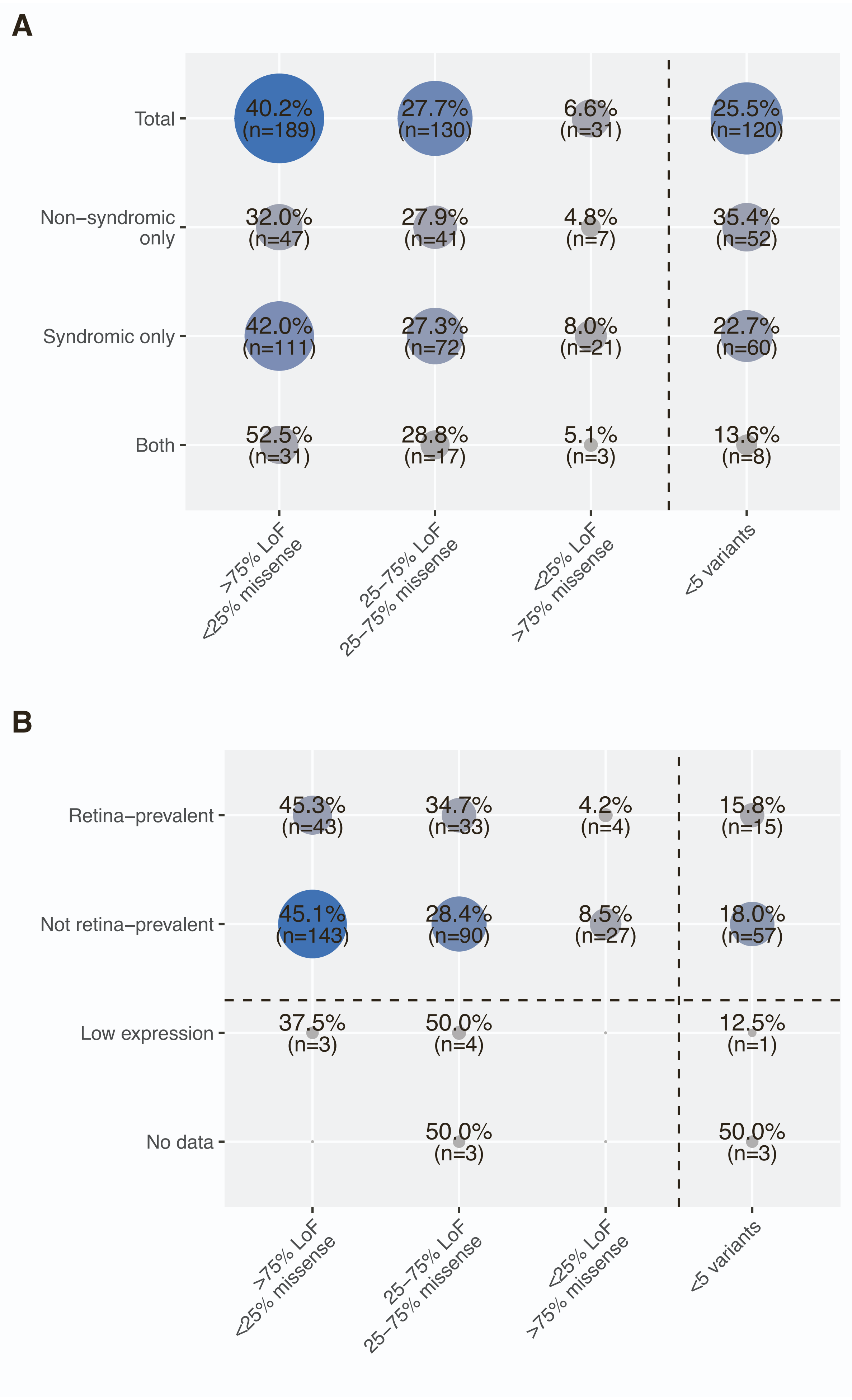

Figure S5: Co-occurrence matrices between types of pathogenic variants and (A) broad phenotypic categories or (B) specific tissue expression from bulk RNA-Seq. n, number of genes.

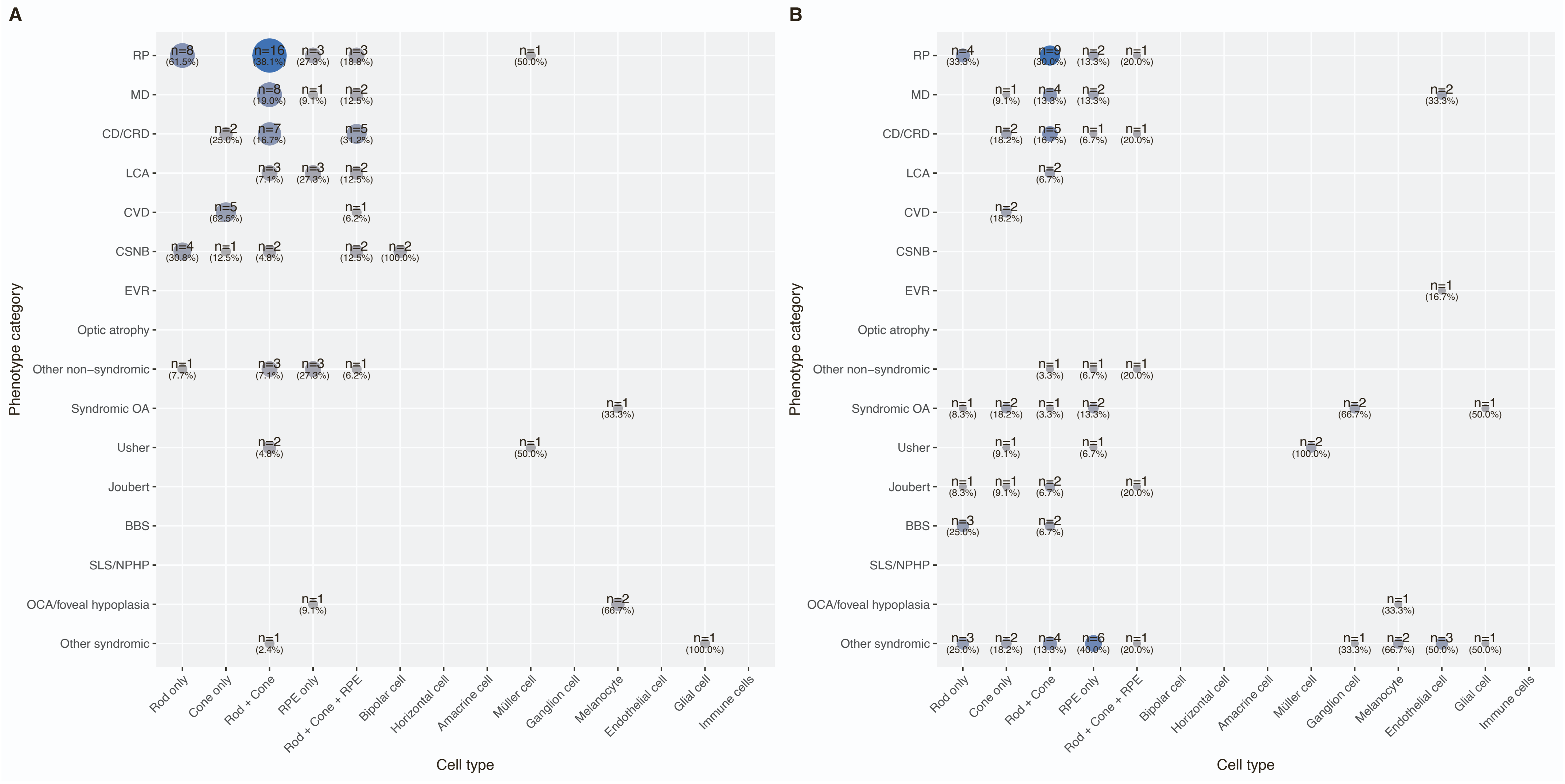

Figure S6: Co-occurrence matrices between phenotypes and scRNAseq data. (A) Retinal-prevalent genes (also minimally expressed in other tissues). (B) Not retinal-prevalent genes, n, number of genes

References

1. Lem, J., Krasnoperova, N.V., Calvert, P.D., Kosaras, B., Cameron, D.A., Nicolo, M., Makino, C.L., and Sidman, R.L. (1999). Morphological, physiological, and biochemical changes in rhodopsin knockout mice. Proc Natl Acad Sci U S A 96, 736-741.
2. Shaikh, R.S., Reuter, P., Sisk, R.A., Kausar, T., Shahzad, M., Maqsood, M.I., Yousif, A., Ali, M., Riazuddin, S., Wissinger, B., et al. (2015). Homozygous missense variant in the human CNGA3 channel causes cone-rod dystrophy. Eur J Hum Genet 23, 473-480.
3. Olichon, A., Baricault, L., Gas, N., Guillou, E., Valette, A., Belenguer, P., and Lenaers, G. (2003). Loss of OPA1 perturbates the mitochondrial inner membrane structure and integrity, leading to cytochrome c release and apoptosis. J Biol Chem 278, 7743-7746.
4. Paes, K.T., Wang, E., Henze, K., Vogel, P., Read, R., Suwanichkul, A., Kirkpatrick, L.L., Potter, D., Newhouse, M.M., and Rice, D.S. (2011). Frizzled 4 is required for retinal angiogenesis and maintenance of the blood-retina barrier. Invest Ophthalmol Vis Sci 52, 6452-6461.
5. Hesse, R.J., Groetsch, J., and Burshell, A. (2010). Pseudoxanthoma Elasticum: A Novel Mutation in the ABCC6 Gene That Affects Eye Manifestations of the Disease. Ochsner J 10, 13-15.
6. ClinGen Consortium. Electronic address, s.b.e., and ClinGen, C. (2025). The Clinical Genome Resource (ClinGen): Advancing genomic knowledge through global curation. Genet Med 27, 101228.
7. Daiger, S.P. (2004). Identifying retinal disease genes: how far have we come, how far do we have to go? Novartis Found Symp 255, 17-27; discussion 27-36, 177-178.
8. Martin, A.R., Williams, E., Foulger, R.E., Leigh, S., Daugherty, L.C., Niblock, O., Leong, I.U.S., Smith, K.R., Gerasimenko, O., Haraldsdottir, E., et al. (2019). PanelApp crowdsources expert knowledge to establish consensus diagnostic gene panels. Nat Genet 51, 1560-1565.
9. Amberger, J.S., Bocchini, C.A., Scott, A.F., and Hamosh, A. (2019). OMIM.org: leveraging knowledge across phenotype-gene relationships. Nucleic Acids Res 47, D1038-D1043.
10. Khan, M., Fadaie, Z., Cornelis, S.S., Cremers, F.P.M., and Roosing, S. (2019). Identification and Analysis of Genes Associated with Inherited Retinal Diseases. Methods Mol Biol 1834, 3-27.
11. Ma, D.J. (2022). Molecular Genetics of Inherited Retinal Diseases. In Inherited Retinal Disease, H.-G. Yu, ed. (Singapore, Springer Nature Singapore), pp 1-19.
12. Henderson, R.H. (2020). Inherited retinal dystrophies. Paediatrics and Child Health 30, 19-27.
13. Bouzidi, A., Charoute, H., Charif, M., Amalou, G., Kandil, M., Barakat, A., and Lenaers, G. (2022). Clinical and genetic spectrums of 413 North African families with inherited retinal dystrophies and optic neuropathies. Orphanet J Rare Dis 17, 197.
14. Tatour, Y., and Ben-Yosef, T. (2020). Syndromic Inherited Retinal Diseases: Genetic, Clinical and Diagnostic Aspects. Diagnostics (Basel) 10.
15. de Bruijn, S.E., Fiorentino, A., Ottaviani, D., Fanucchi, S., Melo, U.S., Corral-Serrano, J.C., Mulders, T., Georgiou, M., Rivolta, C., Pontikos, N., et al. (2020). Structural Variants Create New Topological-Associated Domains and Ectopic Retinal Enhancer-Gene Contact in Dominant Retinitis Pigmentosa. Am J Hum Genet 107, 802-814.
16. Sherman, B.T., Hao, M., Qiu, J., Jiao, X., Baseler, M.W., Lane, H.C., Imamichi, T., and Chang, W. (2022). DAVID: a web server for functional enrichment analysis and functional annotation of gene lists (2021 update). Nucleic Acids Res 50, W216-W221.
17. Lizio, M., Harshbarger, J., Shimoji, H., Severin, J., Kasukawa, T., Sahin, S., Abugessaisa, I., Fukuda, S., Hori, F., Ishikawa-Kato, S., et al. (2015). Gateways to the FANTOM5 promoter level mammalian expression atlas. Genome Biol 16, 22.

18. Uhlen, M., Oksvold, P., Fagerberg, L., Lundberg, E., Jonasson, K., Forsberg, M., Zwahlen, M., Kampf, C., Wester, K., Hober, S., et al. (2010). Towards a knowledge-based Human Protein Atlas. Nat Biotechnol 28, 1248-1250.
19. Cowan, C.S., Renner, M., De Gennaro, M., Gross-Scherf, B., Goldblum, D., Hou, Y., Munz, M., Rodrigues, T.M., Krol, J., Szikra, T., et al. (2020). Cell Types of the Human Retina and Its Organoids at Single-Cell Resolution. Cell 182, 1623-1640 e1634.
20. Wang, K., Li, M., and Hakonarson, H. (2010). ANNOVAR: functional annotation of genetic variants from high-throughput sequencing data. Nucleic Acids Res 38, e164.
