# Supplementary material for: RetiGene, a comprehensive gene atlas for inherited retinal diseases
Source: Am J Hum Genet. 2025 Sep 16;112(10):2253–65. doi: 10.1016/j.ajhg.2025.08.017 (PMC12696501; doi:10.1016/j.ajhg.2025.08.017)
Supplement: Document S2. Article plus supplemental information [file mmc6.pdf]

RetiGene, a comprehensive gene atlas for inherited retinal diseases

Carlo Rivolta,[1,2,3,21,*] Elifnaz Celik,[1,2,21] Dhryata Kamdar,[1,2,21] Francesca Cancellieri,[1,2] Karolina Kaminska,[1,2] Mukhtar Ullah,[1,2] Pilar Barberán-Martínez,[4,5] Manon Bouckaert,[6,7] Marta Cortón,[8,9] Emma Delanote,[6,7] Lidia Fernández-Caballero,[8,9] Gema García García,[4,9] Lara K. Holtes,[10] Marianthi Karali,[11,12] Irma Lopez,[13] Virginie G. Peter,[1,14] Nina Schneider,[15] Lieselot Vincke,[6,7] Carmen Ayuso,[8,9] Sandro Banfi,[11,16] Beatrice Bocquet,[17] Frauke Coppieters,[6,7,18] Frans P.M. Cremers,[10] Chris F. Inglehearn,[19] Takeshi Iwata,[20] Vasiliki Kalatzis,[17] Robert K. Koenekoop,[13] José M. Millán,[4,9] Dror Sharon,[15] Carmel Toomes,[19] and Mathieu Quinodoz[1,2,3,*]

Summary

Inherited retinal diseases (IRDs) are rare disorders, typically presenting as Mendelian traits, that result in stationary or progressive visual impairment. They are characterized by extensive genetic heterogeneity, possibly the highest among all human genetic diseases, as well as diverse inheritance patterns. Despite advances in gene discovery, limited understanding of gene function and challenges in accurately interpreting variants continue to hinder both molecular diagnosis and genetic research in IRDs. One key problem is the absence of a comprehensive and widely accepted catalog of disease-associated genes, which would ensure consistent genetic testing and reliable molecular diagnoses. With the rapid pace of IRD gene discovery, gene catalogs require frequent validation and updates to remain clinically and scientifically useful. To address these gaps, we developed RetiGene, an expert-curated gene atlas that integrates variant data, bulk and single-cell RNA sequencing, and functional annotations. Through the integration of diverse data sources, RetiGene supports candidate gene prioritization, functional studies, and therapeutic development in IRDs.

Introduction

The retina is a photosensitive tissue lining the posterior part of the eye. Its primary function is to convert light into electrical signals, which are then transmitted to the brain to form visual images. The retina contains two types of photoreceptors: rods and cones. Rods are responsible for vision in low-light conditions, while cones provide sharp central vision, enable color perception, and mediate sight in bright-light environments.[1] The retinal pigment epithelium (RPE), a layer of pigmented cells located between the photoreceptors and the choroid, plays a crucial role in supporting vision. It absorbs excess light, forms part of the blood-retina barrier, transports nutrients and waste, regulates the visual cycle, and removes photoreceptor debris, thereby ensuring their proper function.[2,3] Other retinal cell types include retinal ganglion cells, horizontal cells, amacrine cells, and Müller cells, among others, whose function is to encode visual signals detected by photoreceptors and to ensure the correct homeostasis of the retina by providing structural, metabolic, and immunological support.[4–7]

Inherited retinal diseases (IRDs) are a diverse group of monogenic conditions that typically lead to the progressive degeneration or dysfunction of photoreceptors, RPE cells, or other retinal neurons, culminating in vision loss and, in many cases, blindness. Clinically, IRDs are categorized based on the cell types that are first or predominantly affected (e.g., rod-cone degeneration, cone dystrophy, cone-rod degeneration, etc.), the portion of the retina that is primarily involved (center vs. periphery, such as in Stargardt disease and retinitis pigmentosa

[1]Ophthalmic Genetics Group, Institute of Molecular and Clinical Ophthalmology Basel (IOB), Basel 4031, Switzerland; [2]Department of Ophthalmology, University of Basel, Basel 4031, Switzerland; [3]Department of Genetics, Genomics and Cancer Sciences, University of Leicester, Leicester LE1 7RH, UK; [4]Molecular, Cellular, and Genomic Biomedicine Group, IIS-La Fe, Valencia 46026, Spain; [5]Joint Unit CIPF-IIS La Fe Molecular, Cellular and Genomic Biomedicine, IIS-La Fe, Valencia 46026, Spain; [6]Center for Medical Genetics Ghent, Ghent University, Ghent 9000, Belgium; [7]Department of Biomolecular Medicine, Ghent University, Ghent 9000, Belgium; [8]Department of Genetics & Genomics, Instituto de Investigación Sanitaria-Fundación Jiménez Díaz University Hospital, Universidad Autónoma de Madrid (IIS-FJD, UAM), Madrid 28040, Spain; [9]Center for Biomedical Network Research on Rare Diseases (CIBERER), Instituto de Salud Carlos III, Madrid 28029, Spain; [10]Department of Human Genetics, Radboud University Medical Center, Nijmegen, 6525 GA, the Netherlands; [11]Department of Precision Medicine, Medical Genetics, Università degli Studi della Campania "Luigi Vanvitelli", Naples 80138, Italy; [12]Multidisciplinary Department of Medical, Surgical and Dental Sciences, Eye Clinic, Università degli Studi della Campania "Luigi Vanvitelli", Naples 80138, Italy; [13]Department of Paediatric Surgery, Human Genetics, and Ophthalmology, McGill Ocular Genetics Laboratory and Centre, McGill University, Montreal, QC H4A 3S5, Canada; [14]Department of Ophthalmology, Bern University Hospital, Bern 3010, Switzerland; [15]Department of Ophthalmology, Hadassah Medical Center, The Hebrew University of Jerusalem, Jerusalem 91120, Israel; [16]Telethon Institute of Genetics and Medicine, Pozzuoli 80078, Italy; [17]Institute for Neurosciences of Montpellier, Université de Montpellier, Montpellier 34091, France; [18]Department of Pharmaceutics, Ghent University, Ghent 9000, Belgium; [19]Leeds Institute of Medical Research, Division of Molecular Medicine, University of Leeds, Leeds LS2 9JT, UK; [20]Division of Molecular and Cellular Biology, National Institute of Sensory Organs, NHO Tokyo Medical Center, Tokyo 152-8902, Japan
[21]These authors contributed equally
*Correspondence: carlo.rivolta@iob.ch (C.R.), mathieu.quinodoz@iob.ch (M.Q.)
https://doi.org/10.1016/j.ajhg.2025.08.017.
© 2025 The Author(s). Published by Elsevier Inc. on behalf of American Society of Human Genetics.
This is an open access article under the CC BY license (http://creativecommons.org/licenses/by/4.0/).

[RP], respectively), and/or the presence of disease progression (stationary vs. progressive).[8,9] They can also be further classified as non-syndromic (affecting only the eye) or syndromic (affecting the eye along with other organs, such as the auditory or renal systems).[10]

RP is the most prevalent form of IRD, characterized by the degeneration of rods, primarily, and cones, at a later stage,[11,12] whereas cone and cone-rod dystrophies (CDs/CRDs) are characterized by the exclusive or primary loss of cones, respectively.[13,14] The generally stationary forms of cone disorders, grouped as color vision disorders (CVDs), include achromatopsia, blue-cone monochromacy, and common color blindness.[15] Similarly, congenital stationary night blindness (CSNB) is the non-progressive form of rod dysfunction.[16] Macular diseases (MDs), in which degeneration is largely restricted to the macula, include the second most prevalent form of IRDs, Stargardt disease, as well as Best disease, Sorsby macular dystrophy, etc.[12,17,18] Lastly, some other non-syndromic IRDs indirectly affect photoreceptors or involve other retinal cell types, such as optic atrophies (OAs), exudative vitreoretinopathies (EVRs), etc.[19,20] The most severe form of non-syndromic IRDs is Leber congenital amaurosis (LCA), characterized by retinal blindness in early infancy.[21] Syndromic IRDs, though less common, constitute a more heterogeneous group comprising more than 80 described clinical entities. The most prevalent among them are ciliopathies, such as Usher syndrome (USH), Joubert syndrome, Bardet-Biedl syndrome (BBS), and Senior-Løken syndrome (SLS).[12,22,23] Phenotypic variability among patients with the same IRD subtype can also be extensive and include differences in age of onset, rate of progression, severity, etc. Establishing a clinical diagnosis can therefore be a challenging task, often requiring a multidisciplinary approach that combines patient and family medical history with specialized diagnostic tests such as visual acuity and perimetry assessments, electroretinogram (ERG), fundus autofluorescence (FAF), and optical coherence tomography (OCT).[24]

Moreover, despite being monogenic conditions, IRDs are genetically highly heterogeneous and display multiple inheritance patterns (autosomal dominant [AD], autosomal recessive [AR], X-linked, and mitochondrial).[25] Indeed, over 350 genes have been linked to retinal phenotypes, with syndromic forms accounting for ~200 of them and RP alone being associated with ~80 genes.[23,26] Given this genetic complexity, next-generation sequencing (NGS) has become a cost- and time-effective method for the simultaneous screening of multiple genes, especially in large study cohorts. However, the current diagnostic rate, reported in the scientific literature, varies between 53% and 76%, based on results from multiple NGS techniques, such as panel sequencing, whole-exome sequencing (WES), and whole-genome sequencing (WGS).[27–33] This diagnostic gap could be attributed to technical limitations, the existence of genes not yet linked to disease, or variants in regions not typically covered by targeted sequencing procedures, such as intronic or intergenic areas. However, emerging techniques, like *in vitro* RNA splicing assays[34] and long-read sequencing,[35] are being developed to address these challenges.

Another major hurdle in routine molecular diagnosis of IRDs is the use of incomplete or outdated lists of disease-associated genes,[36] which hinders the proper design of real or virtual gene panels, an accurate interpretation of sequencing data, and the establishment of reliable genotype-phenotype associations. In this study, we aim to address this problem by providing an updated list of IRD-related genes, obtained from the latest scientific research, databases of human DNA variations, and repositories of gene expression data. This resource, curated by experts in the field, will be continually updated and made available on a dedicated website, ultimately to help researchers and clinicians identify disease-causing variants and support future discoveries and molecular diagnoses.

Data mining and identification of genes associated with IRDs

Genes associated with IRDs were identified through data mining of public databases and published literature and were individually curated by at least two independent experts, according to the procedures described in the supplemental notes. At the end of the selection process, 470 genes (including four loci: *RP17* [MIM: 600852], *MCDR1* [MIM: 136550], *MCDR3* [MIM: 608850], and Xq27.1 [MIM: 301149]) were retained based on strong evidence of disease association (Figure 1). Another 196 genes were classified as “candidates,” primarily due to evidence from only a single affected family, and 17 genes were excluded due to insufficient evidence, conflicting data, or definitive proof of non-association with IRDs (Table S1).

Phenotypes and inheritance

We chose a two-level approach to phenotype classification. The first level broadly distinguished between syndromic and non-syndromic phenotypes, based on the presence or absence of multisystemic signs in addition to retinal pathology. The second level defined 16 clinical subsets, including 14 specific groups (e.g., RP, MDs, etc.), and two heterogeneous categories that did not fit these groups: “other non-syndromic” and “other syndromic” (Table 1). Notably, we intentionally combined narrowly defined phenotypes such as Stargardt disease, choroideremia, or Sorsby fundus dystrophy into one of the 14 classes, as these entities are each associated with only one or very few genes (e.g., *ABCA4* [MIM: 601691], *CHM* [MIM: 300390], and *TIMP3* [MIM: 188826], respectively).

Out of the 470 curated genes and loci, 206 (202 genes and 4 loci) were associated with non-syndromic diseases

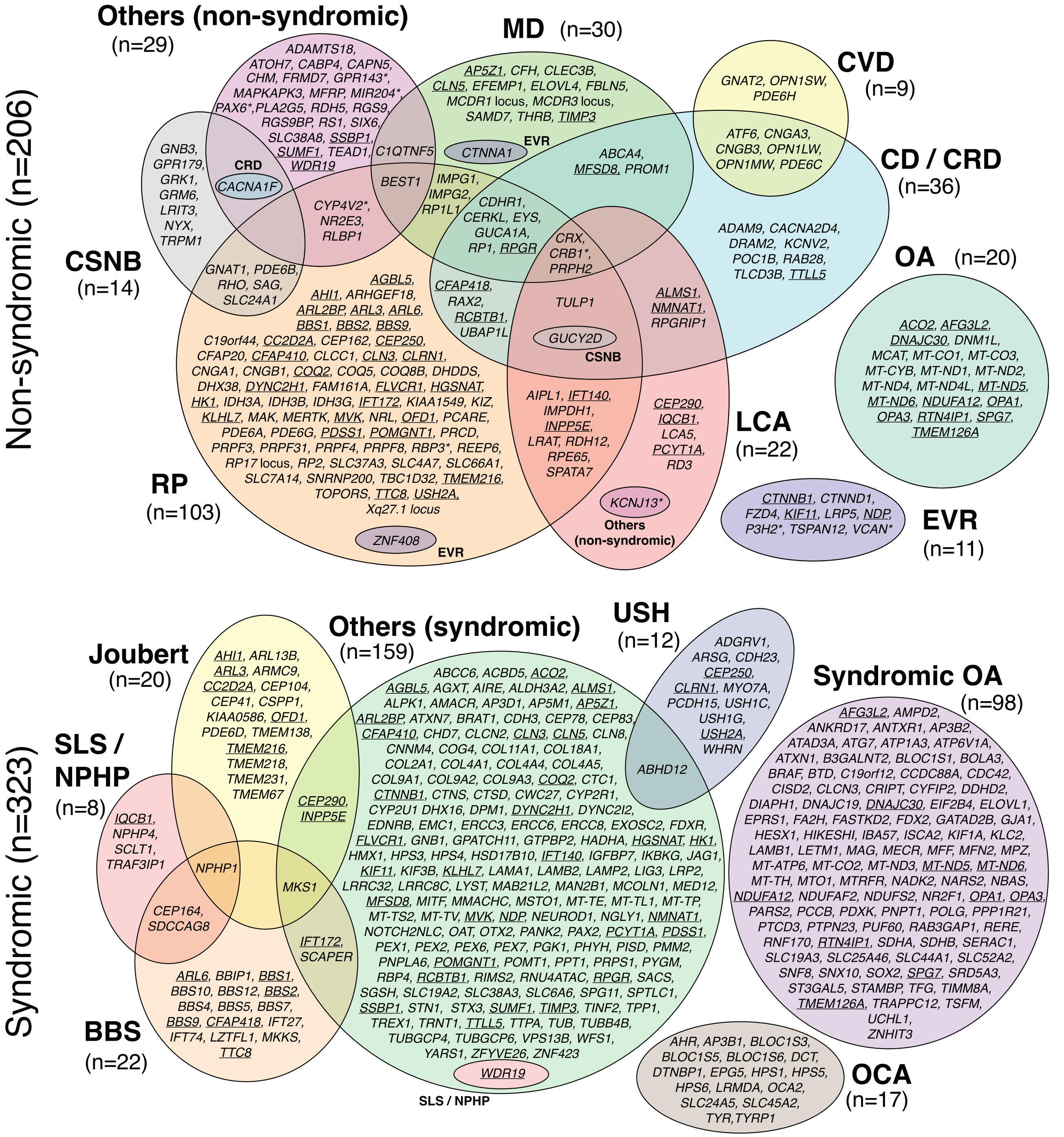

Figure 1. Venn diagram of genes and loci associated with IRDs (total = 470)
Underlined genes are linked to both non-syndromic and syndromic phenotypes. Asterisks point to genes that can also be involved in non-retinal ocular diseases. *n*, number of genes.

in 9 phenotypic groups and 323 were associated with syndromic diseases in 7 phenotypic groups, with 59 genes found to be associated with both non-syndromic and syndromic diseases (Figure 1). Overall, most genes had variants responsible for AR inheritance (68.9%, $n = 324$), followed by AD (15.1%, $n = 71$), AD-AR (7.7%, $n = 36$), X-linked (4.7%, $n = 22$), and mitochondrial (3.6%, $n = 17$) heredity (Table S1; Figure 2).

Among the non-syndromic phenotypes, RP (50.0%, $n = 103$) involved the highest number of genes, followed by CDs/CRDs (17.5%, $n = 36$), MDs (14.6%, $n = 30$), LCA (10.7%, $n = 22$), OAs (9.7%, $n = 20$), CSNB (6.8%, $n = 14$), EVRs (5.3%, $n = 11$), and CVDs (4.4%, $n = 9$), while 29 (14.1%) genes were associated with other non-syndromic phenotypes (Figure 1). Notably, within these genes, 52 (25.2%) were associated with more than one

Table 1. **Clinical classification of various inherited retinal diseases**

Phenotypes and abbreviations	Broad category
Achromatopsia, color vision abnormalities, color blindness (CVD)	non-syndromic
Bardet-Biedl syndrome (BBS)	syndromic
Cone dystrophy, cone-rod dystrophy, Stargardt disease (CD/CRD)	non-syndromic
Congenital stationary night blindness (CSNB)	non-syndromic
Exudative vitreoretinopathy, Norrie disease (EVR)	non-syndromic
Joubert syndrome (Joubert)	syndromic
Leber congenital amaurosis (LCA)	non-syndromic
Macular dystrophy (MD)	non-syndromic
Oculocutaneous albinism, foveal hypoplasia (OCA)	syndromic
Optic atrophy, optic nerve hypoplasia (OA)	non-syndromic
Retinitis pigmentosa (RP)	non-syndromic
Senior-Løken syndrome, nephronophthisis (SLS/NPHP)	syndromic
Syndromic optic atrophy (syndromic OA)	syndromic
Usher syndrome (USH)	syndromic
Others non-syndromic	non-syndromic
Others syndromic	syndromic

non-syndromic phenotype (Table S1; Figure 1). For instance, *CRX* (MIM: 602225), *CRB1* (MIM: 604210), and *PRPH2* (MIM: 179605) were each linked to four phenotypes/clinical categories: RP, MDs, CDs/CRDs, and LCA; similarly, *GUCY2D* (MIM: 600179) was associated with RP, CDs/CRDs, LCA, and CSNB (Figures 1 and S1). In several instances, the genotype-phenotype relationship depended on the type of variant (e.g., loss of function [LoF] vs. missense] or on variant location within specific protein domains.[37–41] For example, in *CRB1*, LoF variants are typically associated with LCA, while missense variants are more commonly linked to RP or MDs.[38,42] Similarly, disease phenotypes associated with *GUCY2D* vary according to the type and location of the variant. Truncating mutations in the extracellular domain cause LCA, whereas missense changes in the protein kinase domain may result in LCA or CSNB, and variants in other parts of the protein are associated with RP, CSNB, CDs/CRDs, or LCA.[43] Likewise, *RPGR* (MIM: 312610) can be linked to RP or CDs/CRDs, depending on the position of the variant along its primary sequence, in a gradient-dependent manner.[44]

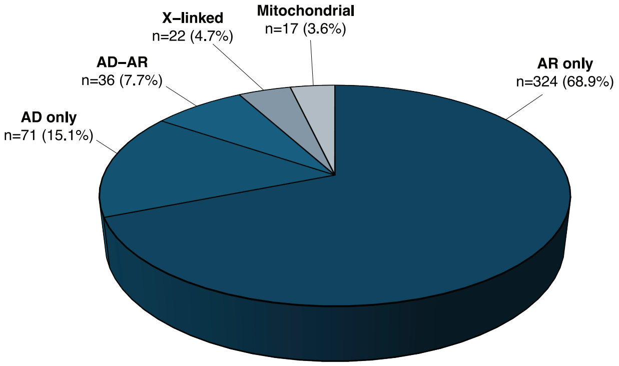

Figure 2. Inheritance mode of diseases associated with all curated genes and loci
n, number of genes.

For syndromic phenotypes, gene associations included syndromic OAs (30.3%, $n = 98$), BBS (6.8%, $n = 22$), Joubert syndrome (6.2%, $n = 20$), USH (3.7%, $n = 12$), SLS/nephronophthisis (NPHP; 2.5%, $n = 8$), oculocutaneous albinism (OCA)/foveal hypoplasia (5.3%, $n = 17$), and “others (syndromic)” (49.2%, $n = 159$), a broad and heterogeneous group involving additional systemic involvement beyond the eye (Figure 1).

Finally, 22 of the 59 genes associated with both syndromic and non-syndromic conditions encoded ciliary proteins, for which severe variants (typically LoF) tend to cause syndromic forms, while milder changes (typically missense or splicing variants) are more often associated with non-syndromic disease (e.g., in *ARL3* [MIM: 604695], *CEP290* [MIM: 610142], or *USH2A* [MIM: 608400]).[45,46]

Historical perspective

Since the first identification of an IRD-associated gene in 1988 (*OAT* [MIM: 613349], linked to gyrate atrophy),[47] the number of genes implicated in these conditions has increased steadily, with an average rate of ~13 discoveries per year (Figure 3A). However, this growth has not been

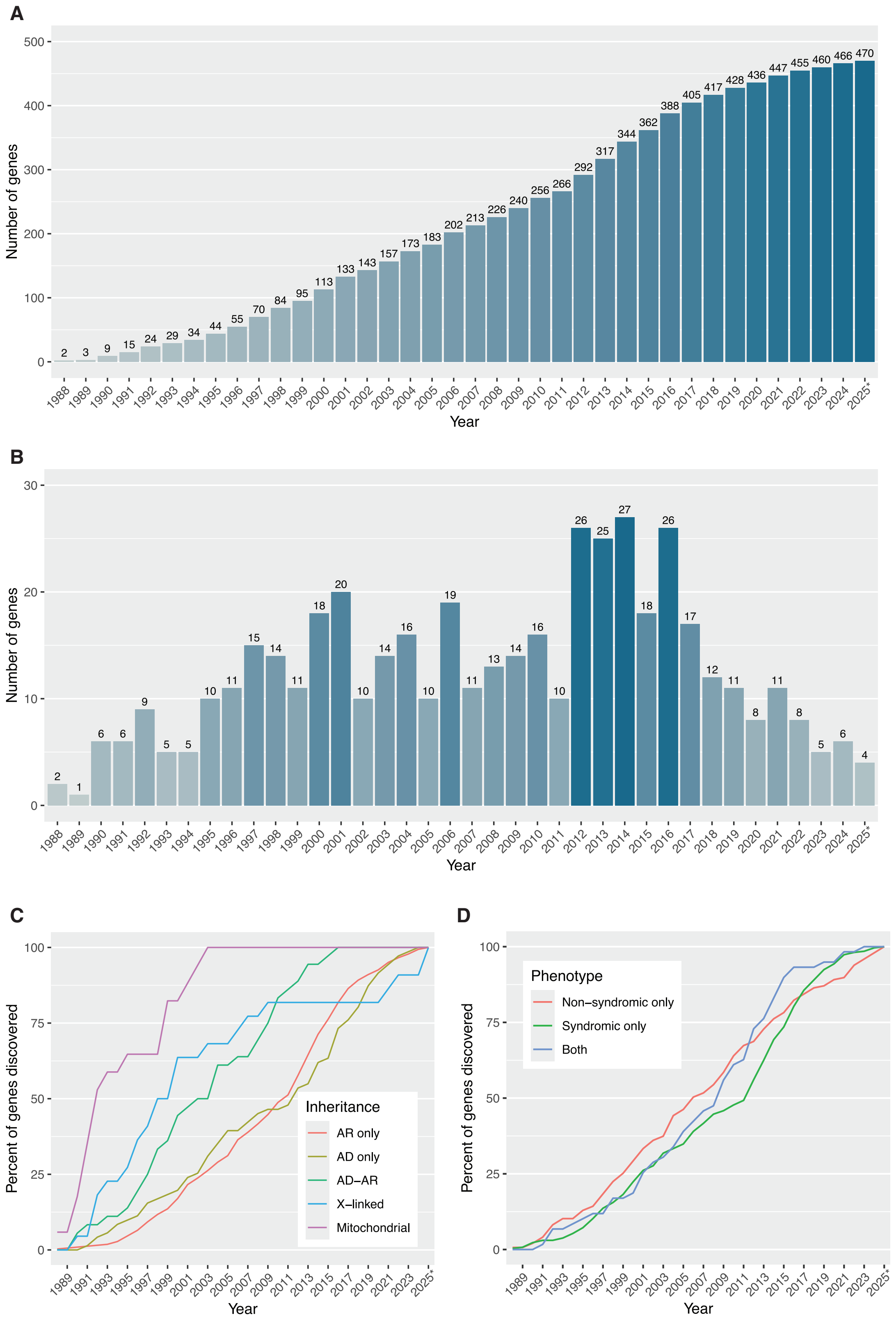

Figure 3. Discovery of IRD genes through time
(A) Cumulative number of genes identified, per year.
(B) Annual count of new gene discoveries.
(C) Cumulative percentage of genes discovered, stratified by inheritance mode.
(D) Cumulative percentage of genes discovered, stratified by broad phenotypic categories.
*As of June 1, 2025.

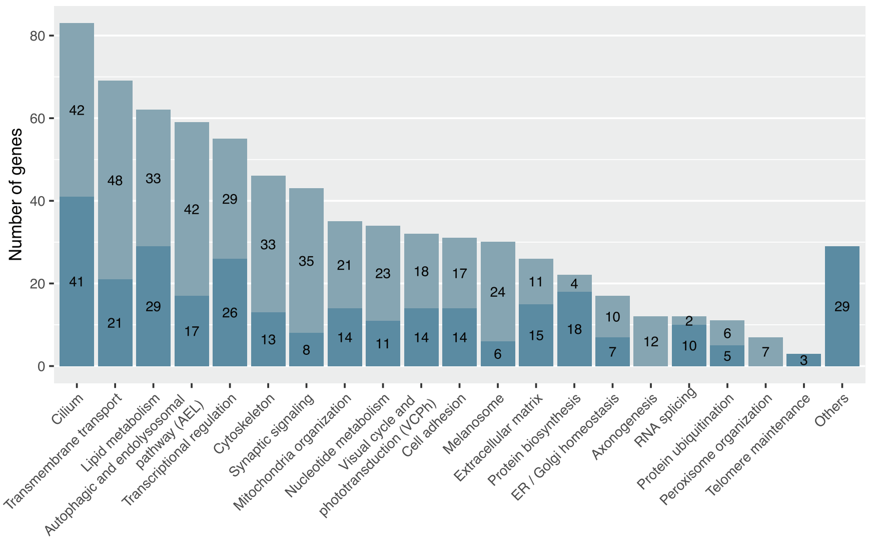

Figure 4. Functional categorization of IRD-associated genes
The bar graph shows the number of genes assigned to each functional category relevant to IRDs. Bar segments indicate whether each gene is annotated in a unique category (blue) or appears in multiple categories (light blue). The "others" group includes genes with roles that could not be confidently assigned to the main categories.

uniform. Until 2010, when gene identification primarily relied on linkage analysis, homozygosity mapping, or candidate gene approaches,[48] the trend was essentially linear, with ~11 new genes identified annually. In 2010, *FAM161A* (MIM: 613596) became the first IRD-associated gene to be identified using NGS,[49] marking the beginning of a more rapid phase of gene discovery, which peaked at ~26 genes per year and lasted until 2018. After 2018, however, the discovery rate declined to ~8 genes per year, with only 6 genes identified in 2024, despite continued access to high-throughput sequencing. This slowdown may reflect the increasing rarity of newly identified genes in terms of gene-specific genetic prevalence, i.e., variants in these genes tend to account for a smaller number of affected individuals in the population (Figure 3B).

When stratifying IRD-associated genes by inheritance mode, we observed that those linked to phenotypes due to mitochondrial DNA defects were usually identified in the earliest years, followed by X-linked genes (Figure 3C), despite mitochondrial and X-linked forms representing the least common inheritance patterns in IRDs (Figure 2). This is likely due to the relative ease of detecting mitochondrial and X-linked inheritance, especially in large pedigrees. In addition, balanced X-autosome translocations in affected females and (micro)deletions in affected males facilitated the positional cloning of several genes on this chromosome (*CHM*, *NDP* [MIM: 300658], *RPGR*, and *RP2* [MIM: 300757]).[50–53] Similarly, although AD inheritance accounts for only 15.1% of all curated genes (Figure 2), AD phenotypes also tended to be discovered earlier than AR phenotypes. This probably reflects the greater statistical power of linkage analysis in AD families compared to AR families of equivalent size (Figure 3C). In contrast, the rate of gene discovery was relatively similar over time between genes associated with non-syndromic and syndromic IRDs (Figure 3D).

Functional classification

We investigated the biological functions of the 466 curated genes (excluding the 4 loci) by stratifying them into 20 functional categories based on Gene Ontology (GO) terms, as detailed in the supplemental notes. Genes not assignable to any of these categories were manually reviewed and grouped under "others". In total, 301 genes (64.6%) fell into a single functional category, while 165 genes (35.4%) were classified into multiple categories (Figure 4). These overlapping classifications enabled the identification of functionally related clusters.

For instance, "cilium" and "cytoskeleton" shared 20 genes, reflecting the cytoskeleton's role as both a structural component of cilia and a regulator of ciliogenesis.[54] Cilium also shared 8 genes with "melanosome," and cytoskeleton shared 7 with "synaptic signaling," due to the involvement of ciliary structures in melanosome transport and actin filaments in synaptic architecture.[55–59] Similarly, "mitochondria organization" and "nucleotide metabolism" overlapped by 10 genes, as several rate-limiting steps of nucleotide metabolism occur in mitochondria.[60] Each of these categories also shared 12–16 genes with "transmembrane transport," related to mitochondrial electron transport processes.[61] "Lipid metabolism" and "visual cycle and phototransduction" (VCPh) shared 9 genes through retinoid metabolism pathways.[62] Furthermore, 10–11 "lipid metabolism" genes overlapped with "autophagic and endolysosomal pathway" (AEL) and "transmembrane transport." Lastly, AEL, synaptic signaling, transmembrane transport, and melanosome shared 7–14 genes, presumably by virtue of their involvement in photoprotection, heterophagy, autophagy in the RPE, and synaptic signal transmission[63,64] (Figure S2).

Cilium was the largest functional category, comprising 83 of the 466 genes (17.8%) (Figure 4). This was expected, given the critical role of cilia in photoreceptor physiology.[65] The second and third largest categories were transmembrane transport (69 genes, 14.8%) and lipid metabolism (62 genes, 13.3%), encompassing proteins not only involved in VCPh but also in membrane-related metabolism that contributes to IRD pathogenesis.

Notably, VCPh ranked tenth, representing only 32 genes (6.9%), reflecting a historical research shift: earlier gene identification efforts specifically targeted VCPh and retina-specific pathways, whereas modern studies are more unbiased.

We next evaluated the distribution of functional categories across three phenotype classes (syndromic, non-syndromic, both) and the 16 clinical subsets described before. In non-syndromic IRDs, the top categories were cilium (37 genes out of 202, 18.3%), transmembrane transport (32 genes, 15.8%), and VCPh (30 genes, 14.9%) (Figure S3A). VCPh was almost exclusively retina specific: 30 of its 32 genes (93.8%) caused non-syndromic IRDs, while other functional classes showed broader phenotypic associations, with less than 25% of their genes confined to non-syndromic cases.

Cilium and VCPh genes were most commonly associated with RP, while transmembrane transport genes were linked equally to RP and OA (Figure S3B). OA-associated genes also belonged to mitochondria and nucleotide metabolism categories, which primarily contribute to OA. CSNB was mainly associated with synaptic signaling, and VCPh genes were consistent with defects in the phototransduction cascade and ribbon synapses. EVR was predominantly linked to “extracellular matrix,” “cell adhesion,” and “transcriptional regulation” genes. Although VCPh genes represented only 30 of the 202 entries associated with non-syndromic IRDs, they were associated with the broadest range of clinical subtypes—including LCA, MDs, CVDs, and CRDs (Figure S3B).

In syndromic IRDs, cilium dominated again (69 genes, 21.4%), reflecting the multisystem involvement typical of ciliopathies (Figure S3A). USH, Joubert syndrome, SLS, and BBS were mainly linked to cilium and cytoskeleton categories (Figure S3C). OCA was represented mainly by melanosome genes, highlighting the dual role of melanin pathways in ocular and cutaneous pigmentation.[66] Syndromic OA was associated with nearly all categories, consistent with its complex etiology. In contrast, as mentioned, VCPh was underrepresented in syndromic IRDs, with only two implicated genes (0.6%), both involved in multiple pathways: *GNB1*, which causes a neurological phenotype,[67] and *RBP4*, associated with skin involvement[68] (Figure S3C).

Inheritance of disease and variant classes

Details about inheritance and phenotypes are shown in Figures 2 and 5A. The majority of curated genes were associated with an AR inheritance pattern across syndromic and non-syndromic phenotypes. AR-associated genes were more frequently linked to purely syndromic presentations (63.0%, $n = 204$) than to non-syndromic ones (24.1%, $n = 78$). AD inheritance was slightly more common in genes associated with syndromic conditions (56.3%, $n = 40$) than with non-syndromic ones (36.6%, $n = 26$), though the difference was modest, and AD inheritance was not observed for prevalent conditions such as USH, Joubert syndrome, BBS, etc. (Figure S4). In contrast, the discrepancy was more pronounced among genes associated with both AD and AR inheritance, with 25 (69.4%) linked to non-syndromic diseases vs. only 4 (11.1%) associated with syndromic conditions. X-linked IRDs were evenly represented across syndromic and non-syndromic forms, as well as specific clinical subtypes. Mitochondrial inheritance was observed in both phenotype classes but was almost exclusively associated with OA. This condition results from the degeneration of RGCs, which transmit visual signals to the brain via the optic nerve and rely on high levels of ATP, a process critically dependent on intact mitochondrial function.[19] Details on the inheritance of syndromic vs. non-syndromic phenotypes are shown in Figure S4.

Next, we investigated whether the spectrum of pathogenic and likely pathogenic (PLP) variants correlates with inheritance mode or phenotypes. Each IRD-associated gene was assigned to one of four categories, based on ClinVar[69] data: (1) > 75% LoF variants, (2) 25%–75% LoF and missense variants (mixed), (3) > 75% missense variants, or (4) fewer than 5 PLP variants (rare). We observed a strong correlation between types of variants and inheritance patterns. Genes associated with AR phenotypes were mostly enriched for LoF variants, followed by mixed or rare classes (Figure 5B). This is consistent with the haplosufficiency of most recessive alleles and the fact that AR conditions typically result from complete protein loss. By contrast, AD conditions generally result from heterozygous gain-of-function, dominant-negative, or haploinsufficient mutations. Accordingly, genes in the AD group were nearly evenly distributed across all four variant categories.[70] Likewise, genes with AD-AR or X-linked inheritance, neither strictly dominant nor recessive, were most commonly found in the mixed LoF/missense group (Figure 5B). LoF variants were also the most prevalent type of DNA changes overall and mostly associated with syndromic conditions and ubiquitously expressed genes (Figure S5).

Gene expression

To investigate a potential correlation between tissue-specific gene expression and the syndromic or non-syndromic nature of retinal disease, we analyzed bulk RNA sequencing (RNA-seq) data from various tissues using the FANTOM5 dataset.[71] Based on gene expression levels in retinal vs. non-retinal tissues, we defined four categories: “retina-prevalent” for genes with significantly higher expression in the retina compared to other tissues, “not retina-prevalent” for genes with expression in the retina but also in other tissues, and “low retinal expression” or “no data” for genes with very low expression in the retina or that were absent from the FANTOM5 dataset, respectively (see supplemental notes).

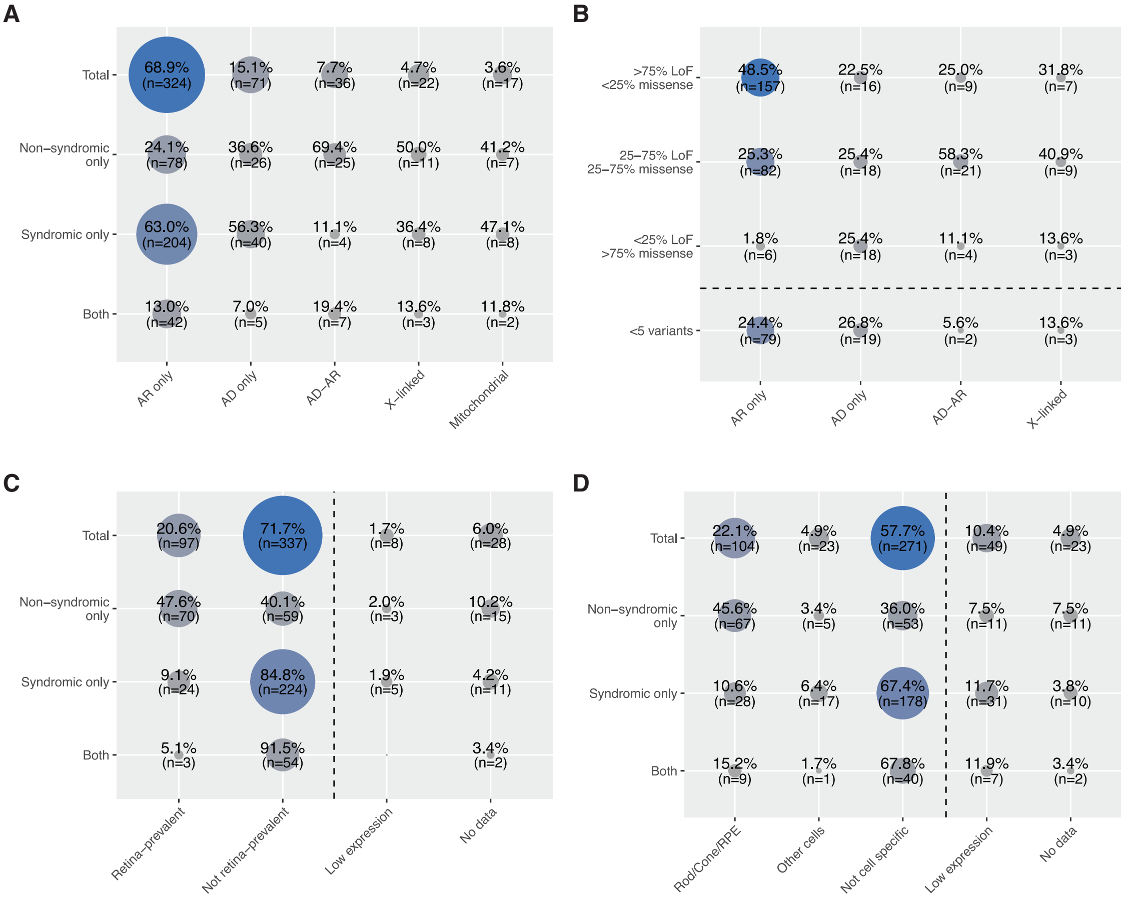

Figure 5. Inheritance and expression features of IRD genes
Co-occurrence matrices between (A) inheritance mode and broad phenotypic categories, (B) inheritance mode and type of pathogenic variants, (C) expression in retina/other tissues and broad phenotypic categories, and (D) expression in retinal cell types and broad phenotypic categories. *n*, number of genes.

We found that 47.6% ($n = 70$) of genes linked only to non-syndromic IRDs were classified as retina prevalent, which aligns well with classical mechanisms of pathogenesis. Conversely, 40.1% ($n = 59$) of genes associated with diseases restricted to the retina were ubiquitously expressed across tissues (Figure 5C). This is not surprising, as many housekeeping genes, essential for all cells, have been previously implicated in non-syndromic IRDs. These include splicing factor genes and genes involved in core metabolic pathways such as the TCA cycle, coenzyme Q biosynthesis, and nucleotide metabolism.[72–75] A widely accepted hypothesis for this paradox is the retina's intrinsic sensitivity to even minimal metabolic or functional disturbances, making it particularly vulnerable compared to other tissues or organs. Supporting this, the majority of genes implicated in syndromic IRDs (84.8%, $n = 224$) or involved in both syndromic and non-syndromic forms (91.5%, $n = 54$) were "not retina-prevalent," many of which are linked to ciliopathies (Figure 5C).

We also examined cell-specific gene expression within the retina in relation to disease phenotype. Using single-cell RNA-seq (scRNA-seq) data, we first classified IRD-associated genes into five categories: "rod/cone/RPE," "other cells," "not cell specific," "low expression," and "no data" (Figure 5D). Notably, 45.6% ($n = 67$) of genes linked to non-syndromic IRDs showed specific expression in photoreceptors or the RPE, compared to 36.0% ($n = 53$) falling into the "not-cell-specific" group. This supports the established concept that non-syndromic IRDs often result from dysfunction or degeneration of cones, rods, RPE cells, or combinations thereof.[76] This observation is reinforced by the fact that most genes linked to syndromic IRDs (67.4%, $n = 178$) showed broad expression across multiple retinal cell types, reflecting their functional relevance in other organs as well. Interestingly, over 15.3% of IRD genes had either minimal or undetectable expression in retinal cells. In addition to developmental stage-specific expression, this is likely due to technical limitations of scRNA-seq, particularly transcript dropout events, rather than true biological absence.[77]

We then analyzed scRNA-seq expression patterns in the context of specific clinical phenotypes (Figures 6 and S6). As expected from clinical and electrophysiological studies, RP was linked to genes expressed in photoreceptors and RPE cells. The involvement of rod-specific genes matches the typical clinical course: initial night blindness followed

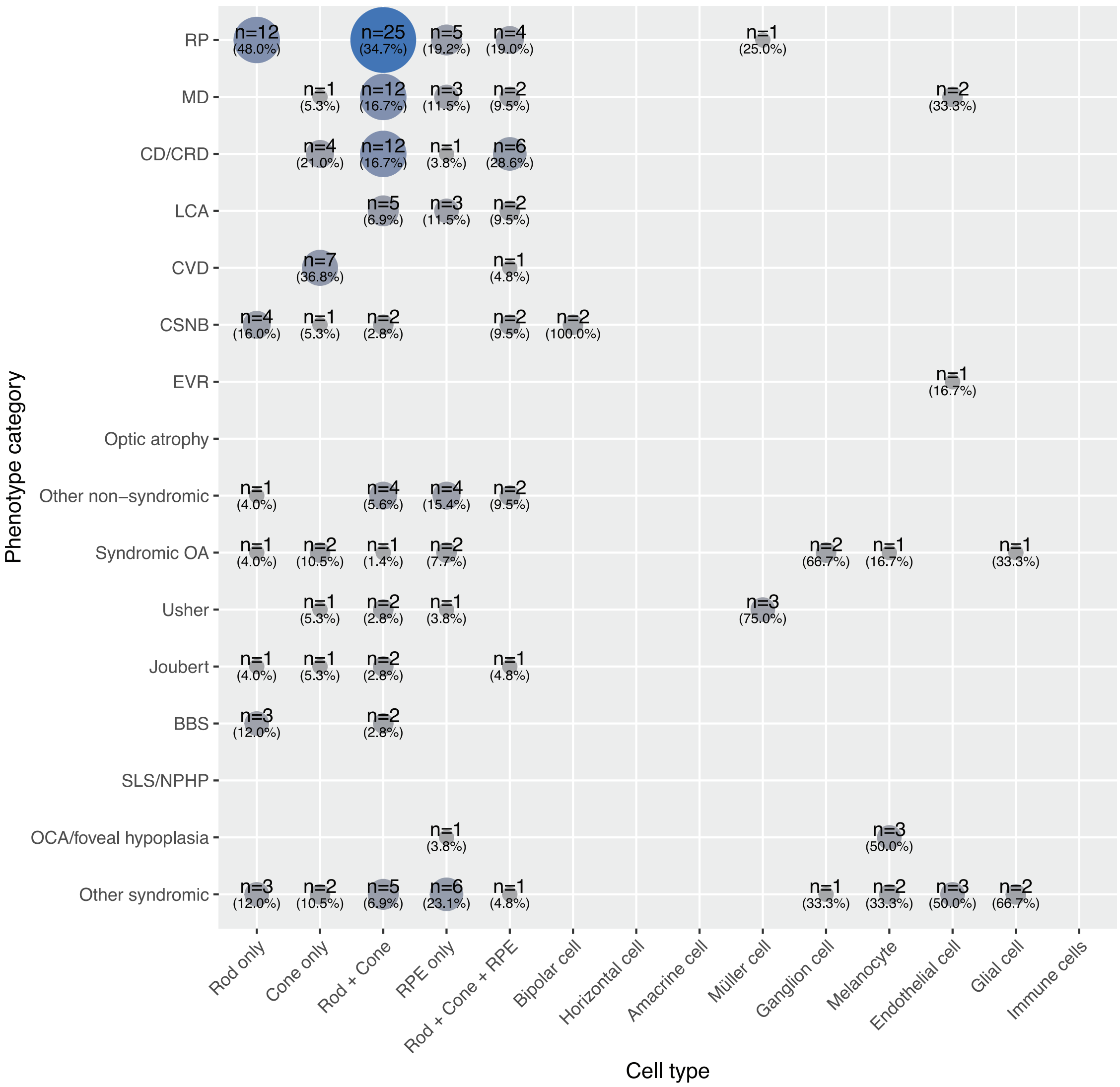

Figure 6. Co-occurrence matrix between phenotypes and retinal single-cell gene expression data
n, number of genes.

by progressive rod and cone degeneration, culminating in tunnel vision. Macular dystrophies and CDs or CRDs could not be reliably distinguished based on scRNA-seq alone, likely due to overlapping expression profiles in the same cell types and differences primarily in the affected retinal regions (macula vs. the entire retina). Interestingly, CDs/CRDs showed a strong involvement of genes expressed exclusively in cones, consistent with cone-only disease mechanisms. LCA, a condition characterized by early-onset, severe vision loss, involved genes affecting rods, cones, and/or the RPE (but not restricted to a single photoreceptor type).[78] This suggests that LCA results from disruptions to fundamental cellular processes necessary for the function of all photoreceptors and the RPE. In CVDs, gene expression was limited to cones, which is consistent with the fact that color vision depends entirely on this photoreceptor subtype. CSNB is associated with nyctalopia from birth and is typically caused by mutations in genes involved in synaptic junctions between photoreceptors (primarily rods) and bipolar cells, although some CSNB subtypes may also involve milder cone dysfunction, as indicated by our analysis (Figures 6 and S6).

The scRNA-seq expression patterns for syndromic IRDs were more diverse. For instance, genes implicated in OCA and foveal hypoplasia were mostly expressed in melanocytes, consistent with their role in melanin biosynthesis. Conversely, some genes associated with syndromic OA were expressed in melanocytes, retinal ganglion, and glial cells, while three USH genes showed expression in Müller cells. Notably, *USH1G* (MIM: 607696), *CLRN1* (MIM: 606397), and *CDH23* (MIM: 605516), linked to USH, have been shown to be involved in the disease through Müller cell dysfunction as well.[79–81] Finally, the group of other syndromic phenotypes involved genes expressed in a wide range of retinal cell types. This broad expression likely reflects real biological relevance rather than an artifact of phenotype grouping. In contrast, genes

associated with non-syndromic conditions (although more numerous overall) tended to show restricted expression limited to photoreceptors and the RPE (Figure 6, top).

Comparison with existing resources and diagnostic relevance

An important strength of RetiGene is its active refinement of content to improve clinical diagnostic accuracy. Compared to existing databases, RetiGene includes a greater number of IRD-associated genes, particularly those linked to syndromic forms, as well as a relatively high proportion of candidate genes (Table S2). These differences likely reflect the effects of manual expert curation combined with stricter inclusion criteria (see supplemental notes).

Such criteria also enable the systematic exclusion of genes with strong contradictory evidence, which is particularly relevant in diagnostic workflows. For example, *UNC119* is still present in many IRD gene panels, despite compelling evidence that its association with dominant disease is likely incorrect. This includes the high frequency of reported pathogenic variants in the general population, lack of co-segregation with disease, and the gene's tolerance to truncating variants.[82–86] Using its standardized curation framework, RetiGene has classified *UNC119* as "discarded," providing a clear signal to diagnostic laboratories that variants in this gene should not be considered clinically meaningful.

Implementation of the database

All the data presented here are hosted on a website with an intuitive user interface, ensuring easy navigation and accessibility (https://retigene.erdc.info/). The content will be regularly updated through literature search and user requests to reflect the latest information, maintaining both accuracy and relevance for users.

Conclusions

In summary, we have assembled a freely accessible online database of genes involved in IRDs, which will be continuously updated. This comprehensive catalog is intended to support the identification of disease-causing variants, the development of more accurate genetic testing panels, and a deeper understanding of the molecular mechanisms underlying these conditions. We believe that this resource will contribute to the development of new targeted therapies, improve diagnostic precision, and ultimately enhance patient care for individuals affected by IRDs.

Data and code availability

All curated genes and figures from this study are available on the RetiGene website (https://retigene.erdc.info/).

Acknowledgments

The authors would like to acknowledge the following funding bodies. E.D. was supported by the Ghent University Special Research Fund (BOF22/DOC/229). M.B. and L.V. were supported by the Research Foundation Flanders (1SD8924N to M.B. and 11PS324N to L.V.). L.F.-C. was supported by the Centro de Investigación Biomédica en Red (CIBER). G.G.G. was supported by the Instituto de Salud Carlos III (ISCIII) (CP22/00028 and PI22/01371) and the European Union, through the HORIZON programme (HORIZON-HLTH-2023-TOOL-05-04, BETTER, 101136262). L.K.H. was supported by the Foundation Fighting Blindness Project Program Award (PPA-0622-0841-UCL). C.A. was supported by ISCIII of the Spanish Ministry of Health (PI22/00321), Centro de Investigación Biomédica en Red Enfermedades Raras (CIBERER, 06/07/0036), IIS-FJD BioBank (PT13/0010/0012), the Organización Nacional de Ciegos Españoles (ONCE), the European Regional Development Fund (FEDER), and the University Chair UAM-IIS-FJD of Genomic Medicine. S.B. was supported by Fondazione Telethon (PE00000006 and CUP H93C22000660006-MNESYS). F.C. was supported by the Research Foundation Flanders (G0ACQ24N). F.P.M.C. was supported by the Foundation Fighting Blindness USA (BR-GE-0120-0775-LUMC). C.F.I. and C.T. were supported by the RP Fighting Blindness and Fight for Sight UK (RP Genome Project GR586). R.K.K. was supported by the Montreal Children's Hospital Foundation, the Vision Sciences Research Network (VSRN), the National Institutes of Health (R01 EY030499-01, Dr. Lentz), the Canadian Institutes of Health Research (CIHR), Fighting Blindness Canada (FBC), and Fonds de Recherche du Québec - Santé (FRQS). R.K.K. also participates in the NAC Attack clinical trial, which is funded by the National Institutes of Health via grants UG1EY033286, UG1EY033293, UG1EY033286, and UG1EY033292. J.M.M. is supported by ISCIII (PI22/00213, AC21_2/00022, and FORT23/00021, the latter co-funded by the European Union) and by the Generalitat Valenciana (CIPROM/2023/26). C.R. is supported by the Swiss National Science Foundation (grant no. 204285).

Declaration of interests

The authors declare no competing interests.

Supplemental information

Supplemental information can be found online at https://doi.org/10.1016/j.ajhg.2025.08.017.

Web resources

OMIM, https://www.omim.org
ClinVar, https://www.ncbi.nlm.nih.gov/clinvar/
RetiGene, https://retigene.erdc.info/
RetNet, https://retnet.org/
Genomics England PanelApp Retinal disorders, https://panelapp.genomicsengland.co.uk/panels/307/
CeGaT Eye Diseases panel, https://cegat.com/diagnostics/rare-diseases/eye-diseases
LOVD, https://www.lovd.nl/
ClinGen Retina GCEP, https://clinicalgenome.org/affiliation/40072/

References

1. Hussey, K.A., Hadyniak, S.E., and Johnston, R.J., Jr. (2022). Patterning and Development of Photoreceptors in the Human Retina. Front. Cell Dev. Biol. *10*, 878350.
2. Strauss, O. (2005). The retinal pigment epithelium in visual function. Physiol. Rev. *85*, 845–881.
3. Intartaglia, D., Giamundo, G., and Conte, I. (2022). Autophagy in the retinal pigment epithelium: a new vision and future challenges. FEBS J. *289*, 7199–7212.
4. Masland, R.H. (2012). The neuronal organization of the retina. Neuron *76*, 266–280.
5. Rashid, K., Akhtar-Schaefer, I., and Langmann, T. (2019). Microglia in Retinal Degeneration. Front. Immunol. *10*, 1975.
6. Reichenbach, A., and Bringmann, A. (2020). Glia of the human retina. Glia *68*, 768–796.
7. Fudalej, E., Justyniarska, M., Kasarełło, K., Dziedziak, J., Szaflik, J.P., and Cudnoch-Jędrzejewska, A. (2021). Neuroprotective Factors of the Retina and Their Role in Promoting Survival of Retinal Ganglion Cells: A Review. Ophthalmic Res. *64*, 345–355.
8. Khan, M., Fadaie, Z., Cornelis, S.S., Cremers, F.P.M., and Roosing, S. (2019). Identification and Analysis of Genes Associated with Inherited Retinal Diseases. Methods Mol. Biol. *1834*, 3–27.
9. Berger, W., Kloeckener-Gruissem, B., and Neidhardt, J. (2010). The molecular basis of human retinal and vitreoretinal diseases. Prog. Retin. Eye Res. *29*, 335–375.
10. Sangermano, R., Galdikaité-Brazienė, E., and Bujakowska, K. M. (2023). Non-syndromic Retinal Degeneration Caused by Pathogenic Variants in Joubert Syndrome Genes. Adv. Exp. Med. Biol. *1415*, 173–182.
11. Verbakel, S.K., van Huet, R.A.C., Boon, C.J.F., den Hollander, A.I., Collin, R.W.J., Klaver, C.C.W., Hoyng, C.B., Roepman, R., and Klevering, B.J. (2018). Non-syndromic retinitis pigmentosa. Prog. Retin. Eye Res. *66*, 157–186.
12. Hanany, M., Shalom, S., Ben-Yosef, T., and Sharon, D. (2024). Comparison of Worldwide Disease Prevalence and Genetic Prevalence of Inherited Retinal Diseases and Variant Interpretation Considerations. Cold Spring Harb. Perspect. Med. *14*, a041277.
13. Tsang, S.H., and Sharma, T. (2018). Progressive Cone Dystrophy and Cone-Rod Dystrophy (XL, AD, and AR). Adv. Exp. Med. Biol. *1085*, 53–60.
14. Park, J.H. (2022). Cone Dystrophy/Cone-Rod Dystrophy. In Inherited Retinal Disease, H.-G. Yu, ed. (Singapore: Springer Nature Singapore), pp. 169–173.
15. Georgiou, M., Robson, A.G., Fujinami, K., de Guimarães, T.A. C., Fujinami-Yokokawa, Y., Daich Varela, M., Pontikos, N., Kalitzeos, A., Mahroo, O.A., Webster, A.R., and Michaelides, M. (2024). Phenotyping and genotyping inherited retinal diseases: Molecular genetics, clinical and imaging features, and therapeutics of macular dystrophies, cone and cone-rod dystrophies, rod-cone dystrophies, Leber congenital amaurosis, and cone dysfunction syndromes. Prog. Retin. Eye Res. *100*, 101244.
16. Zeitz, C., Robson, A.G., and Audo, I. (2015). Congenital stationary night blindness: an analysis and update of genotype-phenotype correlations and pathogenic mechanisms. Prog. Retin. Eye Res. *45*, 58–110.
17. Rahman, N., Georgiou, M., Khan, K.N., and Michaelides, M. (2020). Macular dystrophies: clinical and imaging features, molecular genetics and therapeutic options. Br. J. Ophthalmol. *104*, 451–460.
18. Cremers, F.P.M., Lee, W., Collin, R.W.J., and Allikmets, R. (2020). Clinical spectrum, genetic complexity and therapeutic approaches for retinal disease caused by ABCA4 mutations. Prog. Retin. Eye Res. *79*, 100861.
19. Carelli, V., La Morgia, C., and Yu-Wai-Man, P. (2023). Mitochondrial optic neuropathies. Handb. Clin. Neurol. *194*, 23–42.
20. Ghoraba, H.H., Sears, J., and Traboulsi, E.I. (2025). Hereditary Vitreoretinopathies: Molecular Diagnosis, Clinical Presentation and Management. Clin. Exp. Ophthalmol. *53*, 281–291.
21. Tsang, S.H., and Sharma, T. (2018). Leber Congenital Amaurosis. Adv. Exp. Med. Biol. *1085*, 131–137.
22. Janaky, M., and Braunitzer, G. (2025). Syndromic Retinitis Pigmentosa: A Narrative Review. Vision *9*, 7.
23. Tatour, Y., and Ben-Yosef, T. (2020). Syndromic Inherited Retinal Diseases: Genetic, Clinical and Diagnostic Aspects. Diagnostics *10*, 779.
24. Murro, V., Banfi, S., Testa, F., Iarossi, G., Falsini, B., Sodi, A., Signorini, S., Iolascon, A., Russo, R., Mucciolo, D.P., et al. (2023). A multidisciplinary approach to inherited retinal dystrophies from diagnosis to initial care: a narrative review with inputs from clinical practice. Orphanet J. Rare Dis. *18*, 223.
25. Schneider, N., Sundaresan, Y., Gopalakrishnan, P., Beryozkin, A., Hanany, M., Levanon, E.Y., Banin, E., Ben-Aroya, S., and Sharon, D. (2022). Inherited retinal diseases: Linking genes, disease-causing variants, and relevant therapeutic modalities. Prog. Retin. Eye Res. *89*, 101029.
26. Rodriguez-Munoz, A., Aller, E., Jaijo, T., Gonzalez-Garcia, E., Cabrera-Peset, A., Gallego-Pinazo, R., Udaondo, P., Salom, D., Garcia-Garcia, G., and Millan, J.M. (2020). Expanding the Clinical and Molecular Heterogeneity of Nonsyndromic Inherited Retinal Dystrophies. J. Mol. Diagn. *22*, 532–543.
27. Perea-Romero, I., Gordo, G., Iancu, I.F., Del Pozo-Valero, M., Almoguera, B., Blanco-Kelly, F., Carreño, E., Jimenez-Rolando, B., Lopez-Rodriguez, R., Lorda-Sanchez, I., et al. (2021). Genetic landscape of 6089 inherited retinal dystrophies affected cases in Spain and their therapeutic and extended epidemiological implications. Sci. Rep. *11*, 1526.
28. Sharon, D., Ben-Yosef, T., Goldenberg-Cohen, N., Pras, E., Gradstein, L., Soudry, S., Mezer, E., Zur, D., Abbasi, A.H., Zeitz, C., et al. (2020). A nationwide genetic analysis of inherited retinal diseases in Israel as assessed by the Israeli inherited retinal disease consortium (IIRDC). Hum. Mutat. *41*, 140–149.
29. Liu, X., Tao, T., Zhao, L., Li, G., and Yang, L. (2021). Molecular diagnosis based on comprehensive genetic testing in 800 Chinese families with non-syndromic inherited retinal dystrophies. Clin. Exp. Ophthalmol. *49*, 46–59.
30. Weisschuh, N., Obermaier, C.D., Battke, F., Bernd, A., Kuehlewein, L., Nasser, F., Zobor, D., Zrenner, E., Weber, E., Wissinger, B., et al. (2020). Genetic architecture of inherited retinal degeneration in Germany: A large cohort study from a single diagnostic center over a 9-year period. Hum. Mutat. *41*, 1514–1527.
31. Stone, E.M., Andorf, J.L., Whitmore, S.S., DeLuca, A.P., Giacalone, J.C., Streb, L.M., Braun, T.A., Mullins, R.F., Scheetz, T.E., Sheffield, V.C., and Tucker, B.A. (2017). Clinically Focused Molecular Investigation of 1000 Consecutive Families with Inherited Retinal Disease. Ophthalmology *124*, 1314–1331.

32. Peter, V.G., Kaminska, K., Santos, C., Quinodoz, M., Cancellieri, F., Cisarova, K., Pescini Gobert, R., Rodrigues, R., Custódio, S., Paris, L.P., et al. (2023). The first genetic landscape of inherited retinal dystrophies in Portuguese patients identifies recurrent homozygous mutations as a frequent cause of pathogenesis. PNAS Nexus *2*, pgad043.
33. Karali, M., Testa, F., Di Iorio, V., Torella, A., Zeuli, R., Scarpato, M., Romano, F., Onore, M.E., Pizzo, M., Melillo, P., et al. (2022). Genetic epidemiology of inherited retinal diseases in a large patient cohort followed at a single center in Italy. Sci. Rep. *12*, 20815.
34. Sangermano, R., Khan, M., Cornelis, S.S., Richelle, V., Albert, S., Garanto, A., Elmelik, D., Qamar, R., Lugtenberg, D., van den Born, L.I., et al. (2018). ABCA4 midigenes reveal the full splice spectrum of all reported noncanonical splice site variants in Stargardt disease. Genome Res. *28*, 100–110.
35. Nakamichi, K., Van Gelder, R.N., Chao, J.R., and Mustafi, D. (2023). Targeted adaptive long-read sequencing for discovery of complex phased variants in inherited retinal disease patients. Sci. Rep. *13*, 8535.
36. Mustafi, D., Hisama, F.M., Huey, J., and Chao, J.R. (2022). The Current State of Genetic Testing Platforms for Inherited Retinal Diseases. Ophthalmol. Retina *6*, 702–710.
37. Bianco, L., Arrigo, A., Antropoli, A., Saladino, A., Spiga, I., Patricelli, M.G., Bandello, F., Carrera, P., and Battaglia Parodi, M. (2023). PRPH2-Associated Retinopathy: Novel Variants and Genotype-Phenotype Correlations. Ophthalmol. Retina *7*, 450–461.
38. Bujakowska, K., Audo, I., Mohand-Saïd, S., Lancelot, M.E., Antonio, A., Germain, A., Léveillard, T., Letexier, M., Saraiva, J.P., Lonjou, C., et al. (2012). CRB1 mutations in inherited retinal dystrophies. Hum. Mutat. *33*, 306–315.
39. Fujinami-Yokokawa, Y., Fujinami, K., Kuniyoshi, K., Hayashi, T., Ueno, S., Mizota, A., Shinoda, K., Arno, G., Pontikos, N., Yang, L., et al. (2020). Clinical and Genetic Characteristics of 18 Patients from 13 Japanese Families with CRX-associated retinal disorder: Identification of Genotype-phenotype Association. Sci. Rep. *10*, 9531.
40. Peeters, M.H.C.A., Khan, M., Rooijakkers, A.A.M.B., Mulders, T., Haer-Wigman, L., Boon, C.J.F., Klaver, C.C.W., van den Born, L.I., Hoyng, C.B., Cremers, F.P.M., et al. (2021). PRPH2 mutation update: In silico assessment of 245 reported and 7 novel variants in patients with retinal disease. Hum. Mutat. *42*, 1521–1547.
41. Sharon, D., Wimberg, H., Kinarty, Y., and Koch, K.W. (2018). Genotype-functional-phenotype correlations in photoreceptor guanylate cyclase (GC-E) encoded by GUCY2D. Prog. Retin. Eye Res. *63*, 69–91.
42. Daich Varela, M., Georgiou, M., Alswaiti, Y., Kabbani, J., Fujinami, K., Fujinami-Yokokawa, Y., Khoda, S., Mahroo, O. A., Robson, A.G., Webster, A.R., et al. (2023). CRB1-Associated Retinal Dystrophies: Genetics, Clinical Characteristics, and Natural History. Am. J. Ophthalmol. *246*, 107–121.
43. Rodilla, C., Martín-Merida, I., Blanco-Kelly, F., Trujillo-Tiebas, M.J., Avila-Fernandez, A., Riveiro-Alvarez, R., Del Pozo-Valero, M., Perea-Romero, I., Swafiri, S.T., Zurita, O., et al. (2023). Comprehensive Genotyping and Phenotyping Analysis of GUCY2D-Associated Rod- and Cone-Dominated Dystrophies. Am. J. Ophthalmol. *254*, 87–103.
44. Cehajic-Kapetanovic, J., Martinez-Fernandez de la Camara, C., Birtel, J., Rehman, S., McClements, M.E., Charbel Issa, P., Lotery, A.J., and MacLaren, R.E. (2022). Impaired glutamylation of RPGR(ORF15) underlies the cone-dominated phenotype associated with truncating distal ORF15 variants. Proc. Natl. Acad. Sci. USA *119*, e2208707119.
45. Coppieters, F., Lefever, S., Leroy, B.P., and De Baere, E. (2010). CEP290, a gene with many faces: mutation overview and presentation of CEP290base. Hum. Mutat. *31*, 1097–1108.
46. Gana, S., Serpieri, V., and Valente, E.M. (2022). Genotype-phenotype correlates in Joubert syndrome: A review. Am. J. Med. Genet. C Semin. Med. Genet. *190*, 72–88.
47. Mitchell, G.A., Brody, L.C., Looney, J., Steel, G., Suchanek, M., Dowling, C., Der Kaloustian, V., Kaiser-Kupfer, M., and Valle, D. (1988). An initiator codon mutation in ornithine-delta-aminotransferase causing gyrate atrophy of the choroid and retina. J. Clin. Investig. *81*, 630–633.
48. Dryja, T.P. (1990). Human genetics. Deficiencies in sight with the candidate gene approach. Nature *347*, 614.
49. Langmann, T., Di Gioia, S.A., Rau, I., Stöhr, H., Maksimovic, N.S., Corbo, J.C., Renner, A.B., Zrenner, E., Kumaramanickavel, G., Karlstetter, M., et al. (2010). Nonsense mutations in FAM161A cause RP28-associated recessive retinitis pigmentosa. Am. J. Hum. Genet. *87*, 376–381.
50. Cremers, F.P., van de Pol, D.J., van Kerkhoff, L.P., Wieringa, B., and Ropers, H.H. (1990). Cloning of a gene that is rearranged in patients with choroideraemia. Nature *347*, 674–677.
51. Berger, W., Meindl, A., van de Pol, T.J., Cremers, F.P., Ropers, H.H., Döerner, C., Monaco, A., Bergen, A.A., Lebo, R., Warburg, M., et al. (1992). Isolation of a candidate gene for Norrie disease by positional cloning. Nat. Genet. *1*, 199–203.
52. Meindl, A., Dry, K., Herrmann, K., Manson, F., Ciccodicola, A., Edgar, A., Carvalho, M.R., Achatz, H., Hellebrand, H., Lennon, A., et al. (1996). A gene (RPGR) with homology to the RCC1 guanine nucleotide exchange factor is mutated in X-linked retinitis pigmentosa (RP3). Nat. Genet. *13*, 35–42.
53. Schwahn, U., Lenzner, S., Dong, J., Feil, S., Hinzmann, B., van Duijnhoven, G., Kirschner, R., Hemberger, M., Bergen, A.A., Rosenberg, T., et al. (1998). Positional cloning of the gene for X-linked retinitis pigmentosa 2. Nat. Genet. *19*, 327–332.
54. Mirvis, M., Stearns, T., and James Nelson, W. (2018). Cilium structure, assembly, and disassembly regulated by the cytoskeleton. Biochem. J. *475*, 2329–2353.
55. Williams, D.S., and Lopes, V.S. (2011). The many different cellular functions of MYO7A in the retina. Biochem. Soc. Trans. *39*, 1207–1210.
56. Spence, E.F., and Soderling, S.H. (2015). Actin Out: Regulation of the Synaptic Cytoskeleton. J. Biol. Chem. *290*, 28613–28622.
57. Tayeh, M.K., Yen, H.J., Beck, J.S., Searby, C.C., Westfall, T.A., Griesbach, H., Sheffield, V.C., and Slusarski, D.C. (2008). Genetic interaction between Bardet-Biedl syndrome genes and implications for limb patterning. Hum. Mol. Genet. *17*, 1956–1967.
58. Yen, H.J., Tayeh, M.K., Mullins, R.F., Stone, E.M., Sheffield, V. C., and Slusarski, D.C. (2006). Bardet-Biedl syndrome genes are important in retrograde intracellular trafficking and Kupffer's vesicle cilia function. Hum. Mol. Genet. *15*, 667–677.
59. Jiang, M., Paniagua, A.E., Volland, S., Wang, H., Balaji, A., Li, D.G., Lopes, V.S., Burgess, B.L., and Williams, D.S. (2020). Microtubule motor transport in the delivery of melanosomes to the actin-rich apical domain of the retinal pigment epithelium. J. Cell Sci. *133*, jcs242214.

60. Desler, C., Lykke, A., and Rasmussen, L.J. (2010). The effect of mitochondrial dysfunction on cytosolic nucleotide metabolism. J. Nucleic Acids *2010*, 701518.
61. Pagliarini, D.J., and Rutter, J. (2013). Hallmarks of a new era in mitochondrial biochemistry. Genes Dev. *27*, 2615–2627.
62. Bonet, M.L., Ribot, J., and Palou, A. (2012). Lipid metabolism in mammalian tissues and its control by retinoic acid. Biochim. Biophys. Acta *1821*, 177–189.
63. Boya, P., Kaarniranta, K., Handa, J.T., and Sinha, D. (2023). Lysosomes in retinal health and disease. Trends Neurosci. *46*, 1067–1082.
64. Ivanova, D., and Cousin, M.A. (2022). Synaptic Vesicle Recycling and the Endolysosomal System: A Reappraisal of Form and Function. Front. Synaptic Neurosci. *14*, 826098.
65. Sanchez-Bellver, L., Toulis, V., and Marfany, G. (2021). On the Wrong Track: Alterations of Ciliary Transport in Inherited Retinal Dystrophies. Front. Cell Dev. Biol. *9*, 623734.
66. Hu, D.N., Simon, J.D., and Sarna, T. (2008). Role of ocular melanin in ophthalmic physiology and pathology. Photochem. Photobiol. *84*, 639–644.
67. Nasvytis, M., Ciauskaite, J., and Jurkeviciene, G. (2024). GNB1 Encephalopathy: Clinical Case Report and Literature Review. Medicina (Kaunas) *60*, 589.
68. Steinhoff, J.S., Lass, A., and Schupp, M. (2021). Biological Functions of RBP4 and Its Relevance for Human Diseases. Front. Physiol. *12*, 659977.
69. Landrum, M.J., Lee, J.M., Riley, G.R., Jang, W., Rubinstein, W.S., Church, D.M., and Maglott, D.R. (2014). ClinVar: public archive of relationships among sequence variation and human phenotype. Nucleic Acids Res. *42*, D980–D985.
70. Gerasimavicius, L., Livesey, B.J., and Marsh, J.A. (2022). Loss-of-function, gain-of-function and dominant-negative mutations have profoundly different effects on protein structure. Nat. Commun. *13*, 3895.
71. Lizio, M., Harshbarger, J., Shimoji, H., Severin, J., Kasukawa, T., Sahin, S., Abugessaisa, I., Fukuda, S., Hori, F., Ishikawa-Kato, S., et al. (2015). Gateways to the FANTOM5 promoter level mammalian expression atlas. Genome Biol. *16*, 22.
72. Alcazar-Fabra, M., Rodriguez-Sanchez, F., Trevisson, E., and Brea-Calvo, G. (2021). Primary Coenzyme Q deficiencies: A literature review and online platform of clinical features to uncover genotype-phenotype correlations. Free Radic. Biol. Med. *167*, 141–180.
73. Aweidah, H., Xi, Z., Sahel, J.A., and Byrne, L.C. (2023). PRPF31-retinitis pigmentosa: Challenges and opportunities for clinical translation. Vision Res. *213*, 108315.
74. Brown, E.E., Scandura, M.J., Mehrotra, S., Wang, Y., Du, J., and Pierce, E.A. (2022). Reduced nuclear NAD+ drives DNA damage and subsequent immune activation in the retina. Hum. Mol. Genet. *31*, 1370–1388.
75. Hartong, D.T., Dange, M., McGee, T.L., Berson, E.L., Dryja, T.P., and Colman, R.F. (2008). Insights from retinitis pigmentosa into the roles of isocitrate dehydrogenases in the Krebs cycle. Nat. Genet. *40*, 1230–1234.
76. Manley, A., Meshkat, B.I., Jablonski, M.M., and Hollingsworth, T.J. (2023). Cellular and Molecular Mechanisms of Pathogenesis Underlying Inherited Retinal Dystrophies. Biomolecules *13*, 271.
77. Chen, G., Ning, B., and Shi, T. (2019). Single-Cell RNA-Seq Technologies and Related Computational Data Analysis. Front. Genet. *10*, 317.
78. Cideciyan, A.V. (2010). Leber congenital amaurosis due to RPE65 mutations and its treatment with gene therapy. Prog. Retin. Eye Res. *29*, 398–427.
79. Leong, Y.C., Di Foggia, V., Pramod, H., Bitner-Glindzicz, M., Patel, A., and Sowden, J.C. (2022). Molecular pathology of Usher 1B patient-derived retinal organoids at single cell resolution. Stem Cell Rep. *17*, 2421–2437.
80. Toualbi, L., Toms, M., and Moosajee, M. (2020). USH2A-retinopathy: From genetics to therapeutics. Exp. Eye Res. *201*, 108330.
81. Xu, L., Bolch, S.N., Santiago, C.P., Dyka, F.M., Akil, O., Lobanova, E.S., Wang, Y., Martemyanov, K.A., Hauswirth, W.W., Smith, W.C., et al. (2020). Clarin-1 expression in adult mouse and human retina highlights a role of Muller glia in Usher syndrome. J. Pathol. *250*, 195–204.
82. Kobayashi, A., Higashide, T., Hamasaki, D., Kubota, S., Sakuma, H., An, W., Fujimaki, T., McLaren, M.J., Weleber, R.G., and Inana, G. (2000). HRG4 (UNC119) mutation found in cone-rod dystrophy causes retinal degeneration in a transgenic model. Investig. Ophthalmol. Vis. Sci. *41*, 3268–3277.
83. Li, T., Lin, Y., Gao, H., Chen, C., Zhu, Y., Liu, B., Lian, Y., Li, Y., Zhou, W., Jiang, H., et al. (2017). Two heterozygous mutations identified in one Chinese patient with bilateral macular coloboma. Mol. Med. Rep. *16*, 2505–2510.
84. Hanany, M., and Sharon, D. (2019). Allele frequency analysis of variants reported to cause autosomal dominant inherited retinal diseases question the involvement of 19% of genes and 10% of reported pathogenic variants. J. Med. Genet. *56*, 536–542.
85. Zenteno, J.C., Arce-Gonzalez, R., Matsui, R., Lopez-Bolaños, A., Montes, L., Martinez-Aguilar, A., and Chacon-Camacho, O.F. (2023). Clinical-genetic findings in a group of subjects with macular dystrophies due to mutations in rare inherited retinopathy genes. Graefes Arch. Clin. Exp. Ophthalmol. *261*, 353–365.
86. Chen, S., Francioli, L.C., Goodrich, J.K., Collins, R.L., Kanai, M., Wang, Q., Alföldi, J., Watts, N.A., Vittal, C., Gauthier, L.D., et al. (2024). A genomic mutational constraint map using variation in 76,156 human genomes. Nature *625*, 92–100.

The American Journal of Human Genetics, Volume *112*

Supplemental information

RetiGene, a comprehensive gene atlas for inherited retinal diseases

Carlo Rivolta, Elifnaz Celik, Dhryata Kamdar, Francesca Cancellieri, Karolina Kaminska, Mukhtar Ullah, Pilar Barberán-Martínez, Manon Bouckaert, Marta Cortón, Emma Delanote, Lidia Fernández-Caballero, Gema García García, Lara K. Holtes, Marianthi Karali, Irma Lopez, Virginie G. Peter, Nina Schneider, Lieselot Vincke, Carmen Ayuso, Sandro Banfi, Beatrice Bocquet, Frauke Coppieters, Frans P.M. Cremers, Chris F. Inglehearn, Takeshi Iwata, Vasiliki Kalatzis, Robert K. Koenekoop, José M. Millán, Dror Sharon, Carmel Toomes, and Mathieu Quinodoz

Supplemental Notes

Operational definition

In this study, we define IRDs as conditions that affect the retina directly, such as diseases caused by mutations in *RHO* (MIM: 180380) leading to rod photoreceptor death,[1] *CNGA3* leading to non-functional cones,[2] or *OPA1* (MIM: 605290) leading to ganglion cell death.[3] We also consider conditions that involve the retina secondarily due to pathology originating elsewhere, such as mutations in *FZD4* (MIM: 604579), which cause retinal detachment as a consequence of abnormal retinal vascularization,[4] or in *ABCC6* (MIM: 603234), which is mainly expressed in the liver and leads to abnormal calcium accumulation in Bruch's membrane and its subsequent breakage.[5]

Gene curation

The initial list of genes associated with IRDs was compiled based on data from publicly available sources and then manually curated. The first source was the ClinGen (Retina GCEP, accessed on December 31st, 2024),[6], comprising 119 entries. The second source was RetNet (accessed on January 16, 2025),[7] from which we extracted the full annotated list of IRD genes and loci, totaling 353 entries. The third source was the Genomics England PanelApp (Version 7 of the "Retinal Disorders" panel),[8] which included 430 genes and loci. The fourth source was a IRD-specific list of the Leiden Open Variation Database (LOVD), curated and kindly provided by Prof. Frans Cremers. The fifth source was the CeGaT Eye Disease Panel, including 232 genes as of July 10, 2025. The sixth source was the Online Mendelian Inheritance in Man (OMIM) database (accessed on January 16, 2025),[9] which was queried using the phenotype-specific keywords listed in Table S3, as well as other filters, including: "Gene Map Locus", "Clinical Synopsis", and OMIM's term-specific symbols star (*, genes with known sequence), plus (+, gene with known sequence and phenotype), and dash (−, phenotype with known molecular basis). The combined filtered output initially yielded 1047 entries, from which 609 unique genes were retained after deduplication. The seventh and final source was a structured

literature review using PubMed (accessed on June 1st, 2025), which was also queried with the terms listed in Table S3. To refine search results toward gene-relevant findings, we included Boolean keywords such as “mutation”, “variant”, or “genotype” restricted to the Title or Abstract. All queries were manually reviewed, and a subset of newly associated genes (including *GPATCH11*, *AP5M1*, *AP5Z1*, and *COQ8B*, etc.) was added to the initial list. To ensure completeness, we additionally consulted key review articles.[10-14]

After gathering data from all sources, the gene entries were merged into a master dataset. Following deduplication, the unified list contained 683 unique genes and loci. As of June 1st, 2025, data collection was concluded.

At the end of the collection procedure, each gene was independently evaluated by two experts and included in the downstream analysis if it met either of the following criteria: (i) it harbored distinct pathogenic variants in two or more unrelated individuals or families showing consistent disease phenotype and inheritance pattern; or (ii) it carried the same variant in at least two unrelated individuals or families, supported by strong functional evidence of pathogenicity. Genes that did not fulfill these criteria were classified as “Candidates.” Genes for which conflicting evidence or definitive proof of non-association with IRDs existed were excluded. Loci identified from linkage and association studies without known causative variant(s) were excluded. If they had known causative variants, then they were checked for the criteria of inclusion as stated above. For example, RP17 is a known locus associated with AD-RP due to the presence of complex structural variants which result in ectopic expression of *GDPD1* (MIM: 616317).[15] The RP17 locus was therefore retained since these variants segregated in more than 20 families, thus meeting our criteria of inclusion (i).

Historical perspective selection criteria

Curated genes were annotated with the year of the publication that first linked pathogenic variant(s) in these genes with any form of IRDs.

Functional classification of genes

The 466 curated genes were annotated for biological process GO terms (GOTERM_BP_FAT) using the Functional Annotation tool from the Database for Annotation, Visualization, and Integrated Discovery (DAVID) knowledgebase (version 2021).[16] A total of 446 genes were annotated with over 2500 GO terms in total. Based on these annotations, we grouped the genes into 20 functional categories, as listed in Table S4. Lastly, genes that lacked GO term annotations that fit into these 20 categories were individually assessed by literature review and either manually assigned to one or more of them or grouped into the category "Others". Corresponding PubMed IDs (PMIDs) for these manual annotations are also provided in Table S4.

Manual annotation was also performed for genes that required refinement beyond the DAVID output due to an unspecific GO term assigned. For example, *PDE6B* (MIM: 180072) and *PDE6C* (MIM: 600827) were directly grouped into "visual cycle and phototransduction" functional category based on the GO terms listed in Table S4, while other related genes, such as *PDE6A* (MIM: 180071)*, PDE6G* (MIM: 180073)*, and PDE6H* (MIM: 601190), were annotated under the more generic term "visual perception" (GO:0007601), necessitating their reclassification for consistency across functionally similar genes.

Tissue and retinal cell type expression specificity

To investigate tissue expression of the 470 curated genes and loci in the human transcriptome, the FANTOM5 RNA expression dataset[17] was downloaded from The Human Protein Atlas.[18] The dataset contains normalized transcript-per-million (nTPM) values for 18,287 genes in 60 different human tissue samples, including the retina. The average nTPM values of genes were calculated by grouping some of the tissues as follows: "brain_max" = amygdala, caudate, cerebellum, thalamus, hippocampus, nucleus accumbens, temporal cortex, pituitary gland, putamen, postcentral gyrus, spinal cord, substantia nigra, corpus callosum, frontal lobe, insular cortex, olfactory bulb, pons, occipital pole, occipital lobe, occipital cortex, medulla

oblongata, medial temporal gyrus, medial frontal gyrus; “glands_max” = salivary gland, thyroid gland, thymus; “digestive_max” = colon, esophagus, small intestine, appendix, gallbladder, smooth muscle; “heart” = heart muscle; “liver”; “lung”; “pancreas”; “muscle” = skeletal muscle; “lymph_node” = lymph node; “diversive_max” = prostate, spleen, tongue, urinary bladder, adipose tissue, breast, cervix, endometrium, ovary, vagina, placenta, seminal vesicle. Values from testis, kidney, and retina were not grouped. At the end of this process, tissue samples were organized into 13 distinct sets for downstream analysis.

Then for each gene, the z-score$_{retina}$ (nTPM$_{retina}$ vs nTPM of other tissues) and the expression ratio (ratio$_{retina}$, nTPM$_{retina}$ / nTPM$_{max}$ in other tissues) were calculated using a custom R script. Based on these values, genes were further categorized as follows: “Retina prevalent” = z-score$_{retina}$ > 3 AND ratio$_{retina}$ > 3 AND nTPM$_{retina}$ > 1; “Not retina prevalent” = (z-score ≤ 3 OR ratio$_{retina}$ ≤ 3) AND nTPM$_{retina}$ > 1; “Low expression” = nTPM$_{retina}$ ≤ 1; “No data” = genes that are not included in the FANTOM5 dataset.

Similarly, for the investigation of the single-cell expression of the 470 curated genes within the human retinal tissue, library-normalized transcripts per cell of the adult human peripheral retina were downloaded from a public repository.[19] This dataset contains expression normalized to 10,000 transcript counts per cell type for 57118 genes and 53 cell/cell subtypes of the retina. The 53 cell and cell subtypes were condensed into 19 major groups (Rods, Cones, RPE, Horizontal cells, Amacrine cells, Bipolar Cells, Ganglion cells, Muller cells, Astrocytes, Glial cells, Choroidal melanocyte, Microglial, Monocytes, NK cells, T cells, Mast cells, Pericytes, Fibroblasts, Vascular endothelial cells) by taking the average expression of the cell subtypes. For example, group ‘Cones’ is the average of the L/M and S cone sub-cell types. Further, 5 broader groups were created from these 19 major groups namely, “Rods+Cones” (average of the major groups Rods and Cones), “Rods+Cones+RPE” (average of the major groups Rods, Cones and RPE), “Endothelial cell” (average of major groups Pericytes, Fibroblasts, and Vascular endothelial cells), “Immune cells” (average of major

groups NK cells, T cells, and Mast cells), and “Glial cells” (average of major groups Astrocytes, glial cells and microglial cells).

Then, for each gene, the z-score (normalized expression count of each group vs average of the normalized expression count of remaining groups) and the expression ratio (normalized expression count of each group divided by the average of the normalized expression count of remaining groups) were calculated on a custom R script. Genes were said to be specific to one of the 24 categories (19 major groups and 5 broader groups) based on z-score, ratio, and nTPM as follows: “Group specific” = $z\text{-score}_{group} > 3$ AND $ratio_{group} > 3$ AND $nTPM_{group} > 1$;if they did not meet this criteria for any of the 24 categories, they were marked “Not cell specific”; “Low expression” = $nTPM_{group} \leq 1$; “No data” = genes that are not included in the scRNAseq dataset. For interpretability, we collapsed the results into three overarching specificity categories: “Rods/Cones/RPE” if the gene passed the “Group specific” threshold to be specific to either rods, cones, or RPE, “other cell” if the gene passed the threshold to be specific to either horizontal cells, amacrine cells, bipolar cells, ganglion cell, muller cell, melanocyte, endothelial cell, glial cell or immune cell and “None” if it was found not to be specific to any of the retinal cell groups.

Variant classification

VCF file containing variant information for all the curated genes was downloaded from the ClinVar database (version of 21.01.2023). PLP variants were selected and annotated using ANNOVAR.[20] Then, missense and LoF variants were counted per gene. LoF mutations were defined if the variant led to a canonical splicing event, stopgain mutation, or insertion/deletion leading to a frameshift.

Website maintenance and updates

The results obtained in this work and, specifically, the list of IRD-associated genes, has been made publicly available on a dedicated website (retigene.erdc.info).

Every month, the gene list and the website content will be updated through searches in OMIM and PubMed, as described above. A time filter will be added to review updates from June 1st 2025. Following this search, all genes and articles identified will be manually curated and potentially incorporated into the website. Importantly, we would welcome any feedback from other researchers / users who may directly suggest additions or amendments to the gene list and the website (e.g., about gene associations they have recently discovered or published).

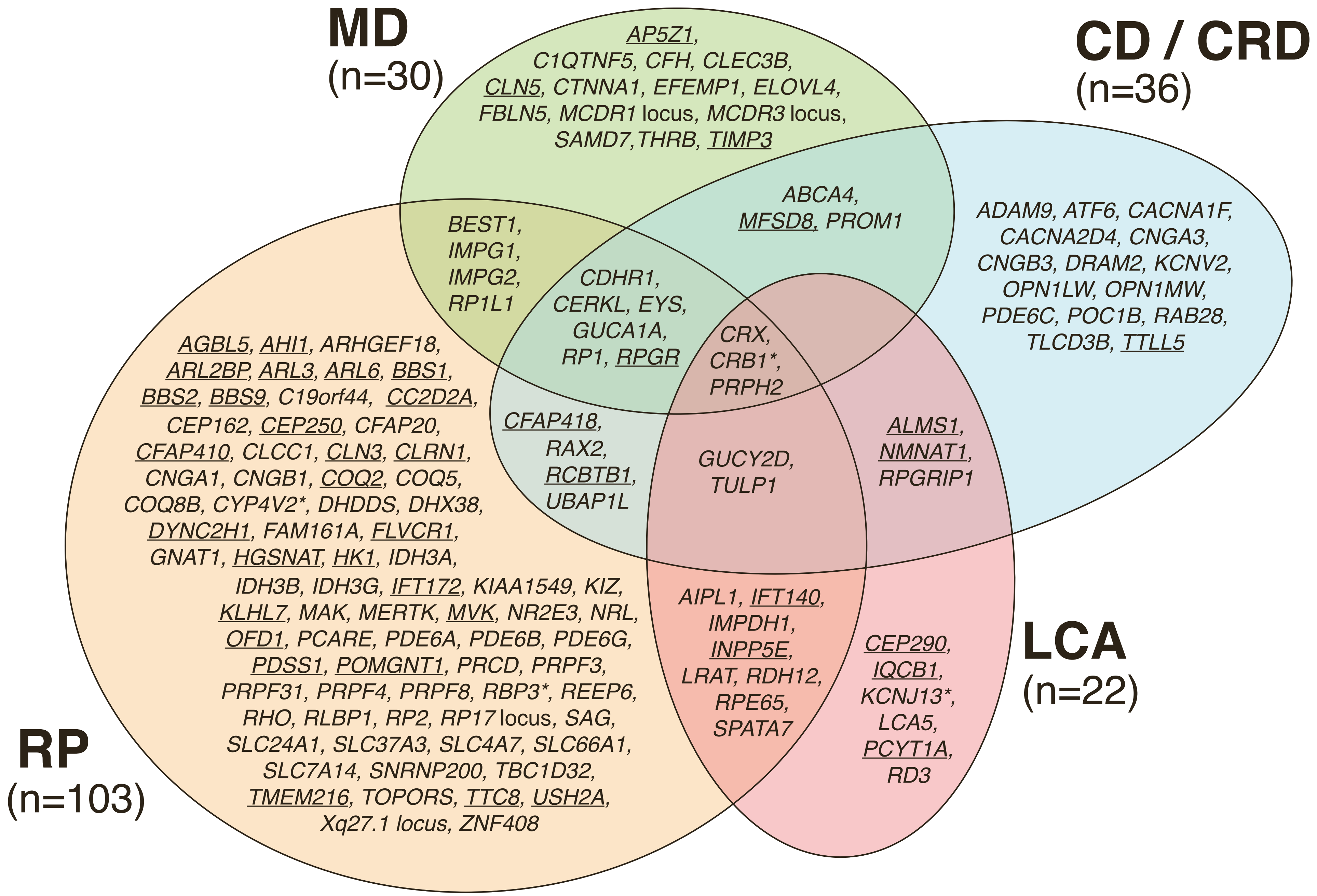

Figure S1: Venn diagram of genes and loci associated with the most common non-syndromic IRDs. Underlined genes are linked to both non-syndromic and syndromic phenotypes. Asterisks point to genes that can also be involved in non-retinal ocular diseases.

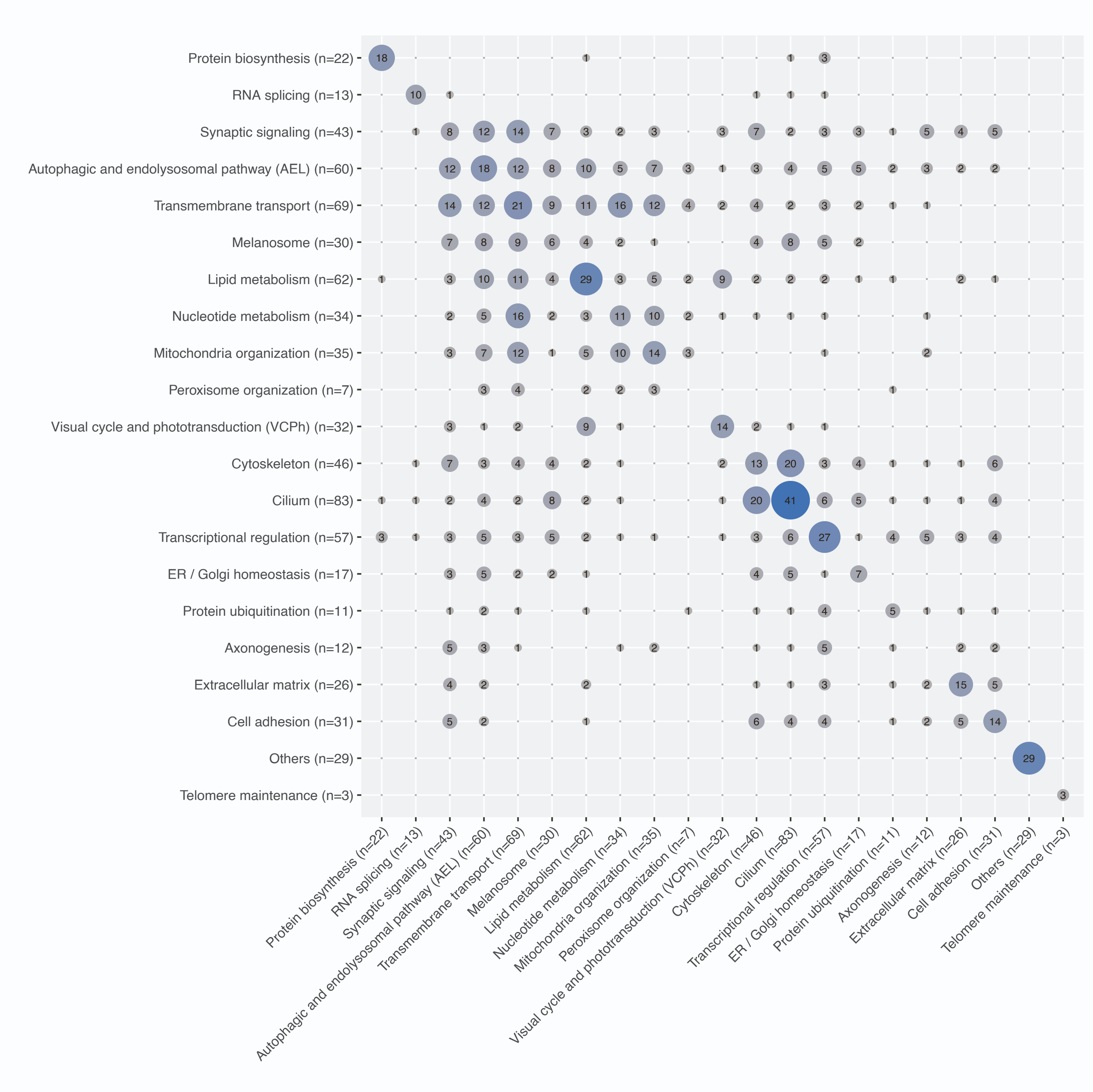

Figure S2. Overlap among functional categories associated with IRD genes. Functional categories are listed along both axes. n (or plain numbers), number of genes.

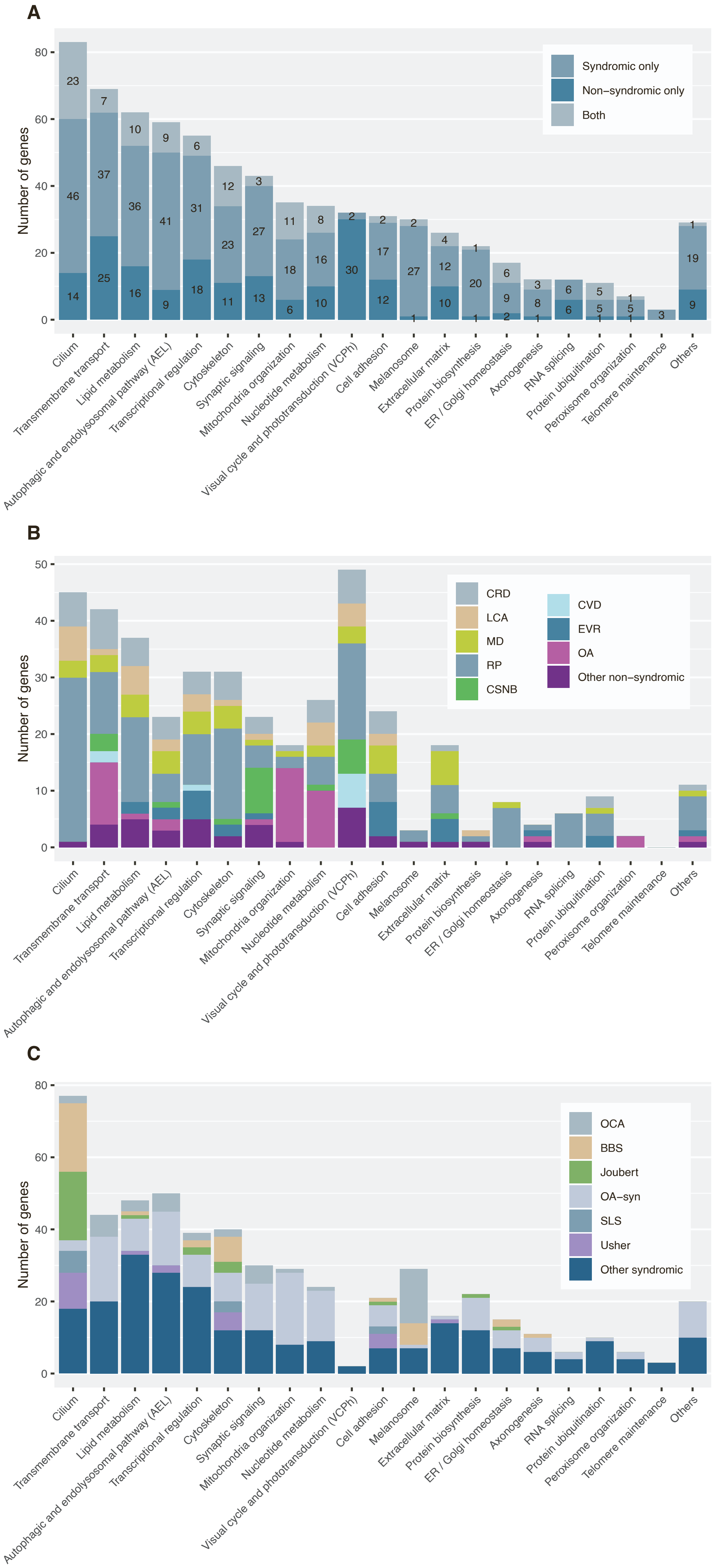

Figure S3: Functional classification of genes, stratified by phenotypes. (A) Broad phenotypic categories. (B) Non-syndromic phenotypes. (C) Syndromic phenotypes.

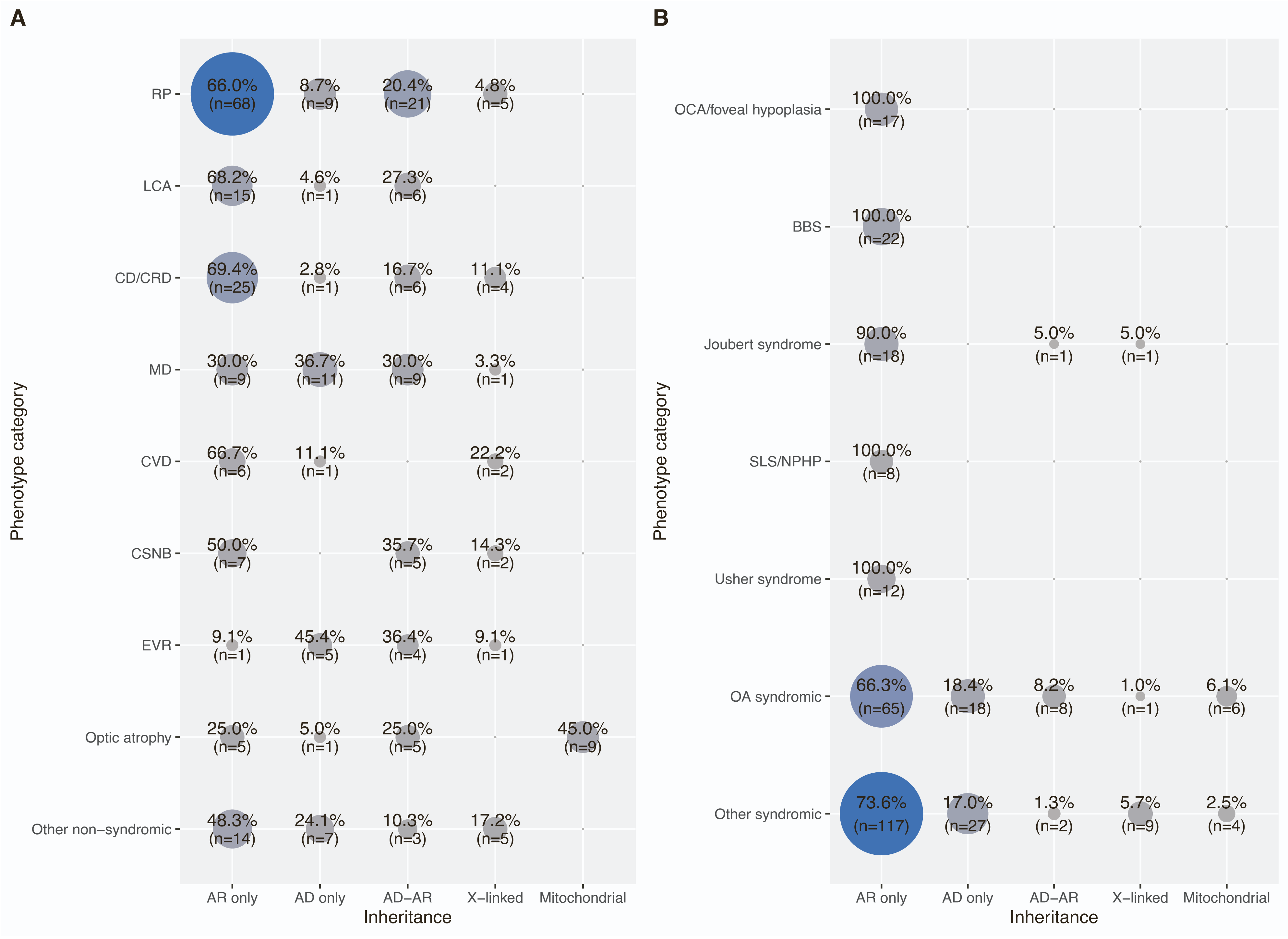

Figure S4: Co-occurrence matrix between phenotypes and their inheritance. (A) Non-syndromic phenotypes. (B) Syndromic phenotypes. n, number of genes.

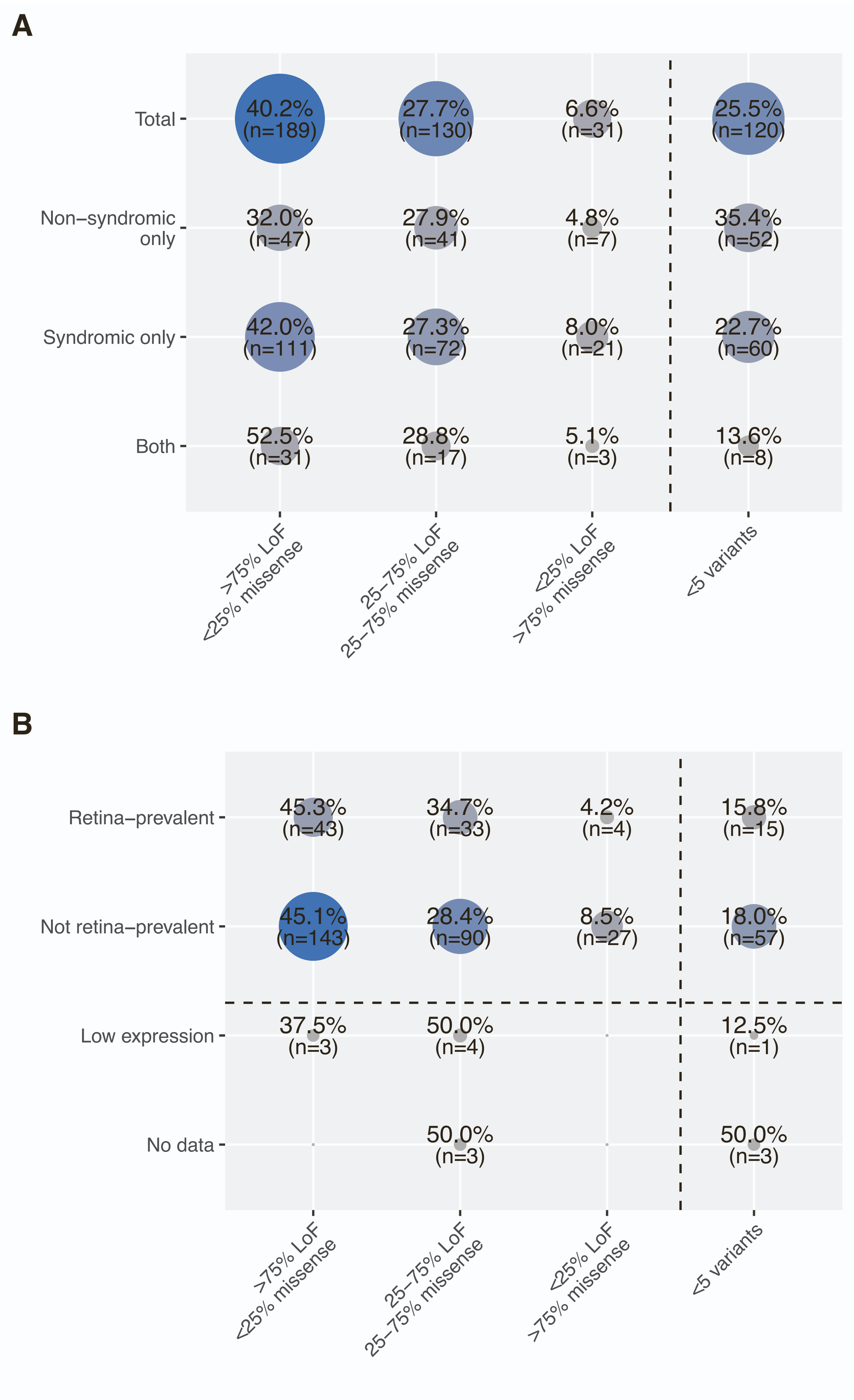

Figure S5: Co-occurrence matrices between types of pathogenic variants and (A) broad phenotypic categories or (B) specific tissue expression from bulk RNA-Seq. n, number of genes.

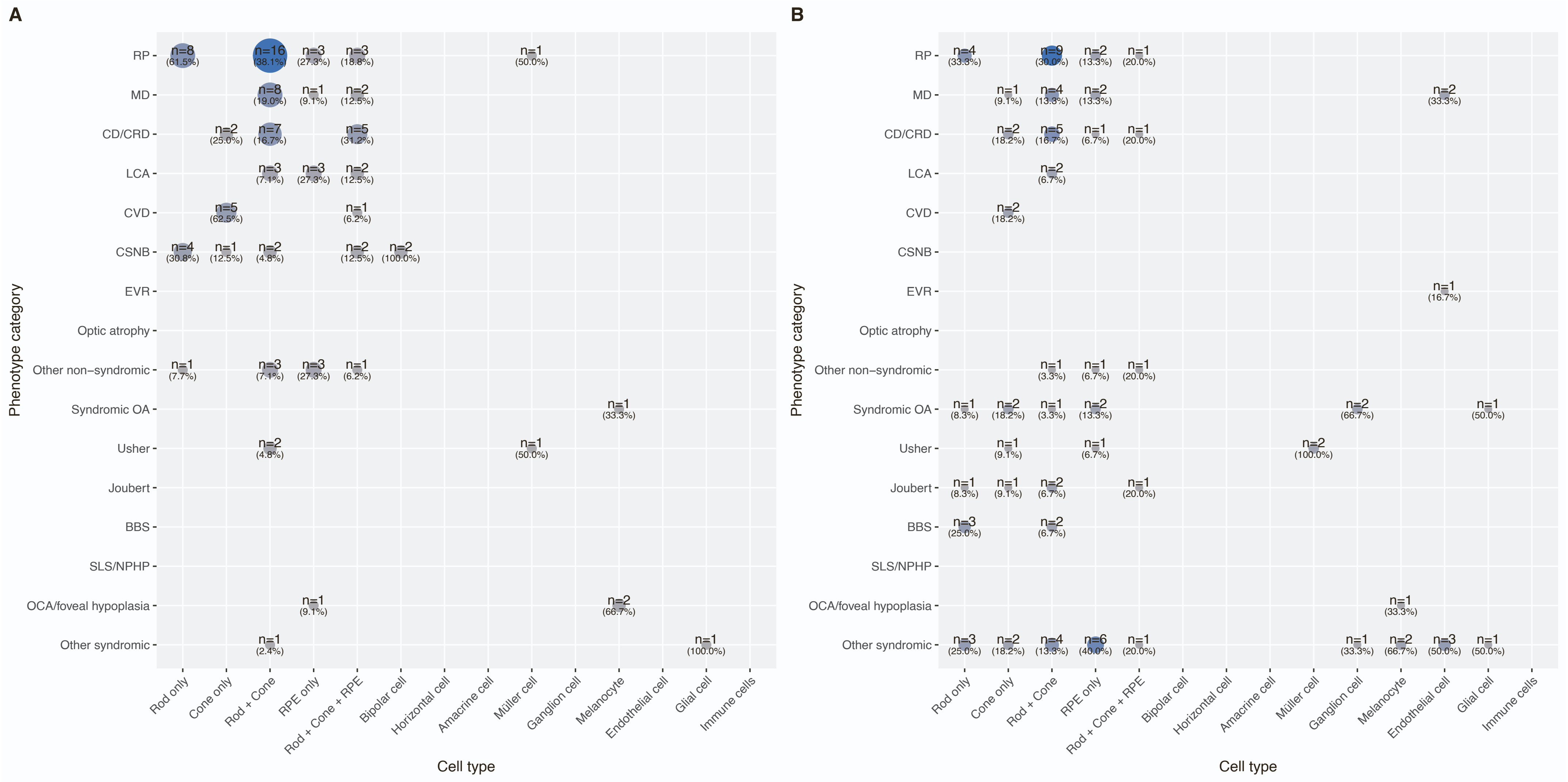

Figure S6: Co-occurrence matrices between phenotypes and scRNAseq data. (A) Retinal-prevalent genes (also minimally expressed in other tissues). (B) Not retinal-prevalent genes, n, number of genes

References

1. Lem, J., Krasnoperova, N.V., Calvert, P.D., Kosaras, B., Cameron, D.A., Nicolo, M., Makino, C.L., and Sidman, R.L. (1999). Morphological, physiological, and biochemical changes in rhodopsin knockout mice. Proc Natl Acad Sci U S A 96, 736-741.
2. Shaikh, R.S., Reuter, P., Sisk, R.A., Kausar, T., Shahzad, M., Maqsood, M.I., Yousif, A., Ali, M., Riazuddin, S., Wissinger, B., et al. (2015). Homozygous missense variant in the human CNGA3 channel causes cone-rod dystrophy. Eur J Hum Genet 23, 473-480.
3. Olichon, A., Baricault, L., Gas, N., Guillou, E., Valette, A., Belenguer, P., and Lenaers, G. (2003). Loss of OPA1 perturbates the mitochondrial inner membrane structure and integrity, leading to cytochrome c release and apoptosis. J Biol Chem 278, 7743-7746.
4. Paes, K.T., Wang, E., Henze, K., Vogel, P., Read, R., Suwanichkul, A., Kirkpatrick, L.L., Potter, D., Newhouse, M.M., and Rice, D.S. (2011). Frizzled 4 is required for retinal angiogenesis and maintenance of the blood-retina barrier. Invest Ophthalmol Vis Sci 52, 6452-6461.
5. Hesse, R.J., Groetsch, J., and Burshell, A. (2010). Pseudoxanthoma Elasticum: A Novel Mutation in the ABCC6 Gene That Affects Eye Manifestations of the Disease. Ochsner J 10, 13-15.
6. ClinGen Consortium. Electronic address, s.b.e., and ClinGen, C. (2025). The Clinical Genome Resource (ClinGen): Advancing genomic knowledge through global curation. Genet Med 27, 101228.
7. Daiger, S.P. (2004). Identifying retinal disease genes: how far have we come, how far do we have to go? Novartis Found Symp 255, 17-27; discussion 27-36, 177-178.
8. Martin, A.R., Williams, E., Foulger, R.E., Leigh, S., Daugherty, L.C., Niblock, O., Leong, I.U.S., Smith, K.R., Gerasimenko, O., Haraldsdottir, E., et al. (2019). PanelApp crowdsources expert knowledge to establish consensus diagnostic gene panels. Nat Genet 51, 1560-1565.
9. Amberger, J.S., Bocchini, C.A., Scott, A.F., and Hamosh, A. (2019). OMIM.org: leveraging knowledge across phenotype-gene relationships. Nucleic Acids Res 47, D1038-D1043.
10. Khan, M., Fadaie, Z., Cornelis, S.S., Cremers, F.P.M., and Roosing, S. (2019). Identification and Analysis of Genes Associated with Inherited Retinal Diseases. Methods Mol Biol 1834, 3-27.
11. Ma, D.J. (2022). Molecular Genetics of Inherited Retinal Diseases. In Inherited Retinal Disease, H.-G. Yu, ed. (Singapore, Springer Nature Singapore), pp 1-19.
12. Henderson, R.H. (2020). Inherited retinal dystrophies. Paediatrics and Child Health 30, 19-27.
13. Bouzidi, A., Charoute, H., Charif, M., Amalou, G., Kandil, M., Barakat, A., and Lenaers, G. (2022). Clinical and genetic spectrums of 413 North African families with inherited retinal dystrophies and optic neuropathies. Orphanet J Rare Dis 17, 197.
14. Tatour, Y., and Ben-Yosef, T. (2020). Syndromic Inherited Retinal Diseases: Genetic, Clinical and Diagnostic Aspects. Diagnostics (Basel) 10.
15. de Bruijn, S.E., Fiorentino, A., Ottaviani, D., Fanucchi, S., Melo, U.S., Corral-Serrano, J.C., Mulders, T., Georgiou, M., Rivolta, C., Pontikos, N., et al. (2020). Structural Variants Create New Topological-Associated Domains and Ectopic Retinal Enhancer-Gene Contact in Dominant Retinitis Pigmentosa. Am J Hum Genet 107, 802-814.
16. Sherman, B.T., Hao, M., Qiu, J., Jiao, X., Baseler, M.W., Lane, H.C., Imamichi, T., and Chang, W. (2022). DAVID: a web server for functional enrichment analysis and functional annotation of gene lists (2021 update). Nucleic Acids Res 50, W216-W221.
17. Lizio, M., Harshbarger, J., Shimoji, H., Severin, J., Kasukawa, T., Sahin, S., Abugessaisa, I., Fukuda, S., Hori, F., Ishikawa-Kato, S., et al. (2015). Gateways to the FANTOM5 promoter level mammalian expression atlas. Genome Biol 16, 22.

18. Uhlen, M., Oksvold, P., Fagerberg, L., Lundberg, E., Jonasson, K., Forsberg, M., Zwahlen, M., Kampf, C., Wester, K., Hober, S., et al. (2010). Towards a knowledge-based Human Protein Atlas. Nat Biotechnol 28, 1248-1250.
19. Cowan, C.S., Renner, M., De Gennaro, M., Gross-Scherf, B., Goldblum, D., Hou, Y., Munz, M., Rodrigues, T.M., Krol, J., Szikra, T., et al. (2020). Cell Types of the Human Retina and Its Organoids at Single-Cell Resolution. Cell 182, 1623-1640 e1634.
20. Wang, K., Li, M., and Hakonarson, H. (2010). ANNOVAR: functional annotation of genetic variants from high-throughput sequencing data. Nucleic Acids Res 38, e164.
